# Maternal Mental Health in Pregnancy and Its Impact on Children’s Cognitive Development at 18 Months, during the COVID-19 Pandemic (CONCEPTION Study)

**DOI:** 10.3390/jcm13041055

**Published:** 2024-02-13

**Authors:** Narimene Ait Belkacem, Jessica Gorgui, Vanina Tchuente, Delphine Aubin, Sarah Lippé, Anick Bérard

**Affiliations:** 1 Faculty of Pharmacy, University of Montreal, Montreal, QC H3T 1J4, Canada; narimene.ait.belkacem@umontreal.ca (N.A.B.); jessica.gorgui@umontreal.ca (J.G.); 2 Research Center, CHU Sainte-Justine, Montreal, QC H3T 1C5, Canada; vanina.tchuente.hsj@ssss.gouv.qc.ca (V.T.); delphine.aubin@umontreal.ca (D.A.); sarah.lippe@umontreal.ca (S.L.); 3 Department of Psychology, University of Montreal, Montreal, QC H2V 2S9, Canada; 4 Faculty of Medicine, Université Claude Bernard, Lyon 1, 69003 Lyon, France

**Keywords:** COVID-19 pandemic, prenatal mental health, in utero exposure, pregnancy, child development, 18 months of age, ASQ-3

## Abstract

Background: The COVID-19 pandemic has significantly affected the mental health of pregnant persons. Objective: We aimed to evaluate the impact of maternal mental health and antidepressant use on children’s cognitive development. Methods: We followed a cohort of children born during the COVID-19 pandemic. Maternal mental health was self-reported during pregnancy (Edinburgh Postnatal Depression Scale, General Anxiety Disorder-7, stress levels, and antidepressant use). The child’s cognitive development was measured using the third edition of the Ages & Stages Questionnaires^®^ (ASQ-3) at 18 months. Multivariate multinomial logistic regression models were built to assess the association between in utero exposure to maternal mental health and ASQ-3 domains: communication, gross motor, fine motor, problem-solving, and personal–social. Results: Overall, 472 children were included in our analyses. After adjusting for potential confounders, a need for further assessment in communication (adjusted odds ratio (aOR) 12.2, 95% confidence interval (CI) (1.60;92.4)), and for improvement in gross motricity (aOR 6.33, 95%CI (2.06;19.4)) were associated with in utero anxiety. The need for improvement in fine motricity (aOR 4.11, 95%CI (1.00; 16.90)) was associated with antidepressant exposure. In utero depression was associated with a decrease in the need for improvement in problem solving (aOR 0.48, 95%CI (0.24; 0.98)). Conclusions: During the COVID-19 pandemic, maternal mental health appears to be associated with some aspects of children’s cognitive development.

## 1. Introduction

Maternal mental health is a pressing public health issue. During pregnancy, depression and anxiety are the most common psychological conditions, with a prevalence of 20.7% (95% confidence interval (CI) (19.4;21.9)) for depressive [1] and 18.2% (95%CI (13.6;22.8)) anxious symptoms [2]. Evidence suggests that the psychological well-being of pregnant persons often deteriorates during critical periods marked by stressful events [3]. Indeed, during the 2003 Severe acute respiratory syndrome (SARS) outbreak in Hong Kong, a study revealed that pregnant persons in the SARS cohort presented significantly higher anxiety state scores compared to the pre-SARS cohort (37.2 ± 9.7 vs. 35.5 ± 9.3, *p* = 0.02) [4]. The COVID-19 pandemic further highlighted this concern. Following the pandemic’s announcement in 2020, studies assessed the impact of measures implemented to curb the spread of the SARS coronavirus 2 (SARS-CoV-2) [5]. Reports indicate a rise in symptoms of depression [6,7], anxiety [8], and stress among pregnant persons during the COVID-19 pandemic [9] compared to the pre-pandemic period, signaling the pandemic’s profound impact on maternal mental health [10]. Moreover, Berard et al., within the CONCEPTION Study, reported that the COVID-19 pandemic affected maternal mental health in pregnancy differently depending on the trimester of gestation [11]. Results suggested that prenatal depression and stress tend to increase progressively throughout pregnancy. Additionally, the study noted an increased score for maternal depression during the second wave of the pandemic (September 2020 to March 2021) compared to the first wave, attributed to reinstated closures and the absence of available vaccines [11]. However, depression mean scores decreased during the third wave, although anxiety symptoms remained largely unchanged [11]. Maternal mental health is intricately linked with prenatal outcomes and early neurodevelopment in children, including cognitive development [12,13]. According to the fifth edition of the diagnostic and statistical manual of mental disorders (DSM-5), the prevalence of neurodevelopmental disorders in childhood ranges from 0.70% to 3% for specific learning disorders and from 3% to 10% for communication disorders, and 0.76–17% for motor disorders in the general population [14]. Koutra et al. reported that prenatal maternal depressive symptoms were linked to decreased cognitive development in children (β coefficient −5.45, 95%CI: (−10.44; −0.46)), assessed with the third edition of the Bayley Scales of Infant and Toddler Development [15]. A recent study by Putnick et al. supports the idea that prenatal depression is associated with any domain of the third edition of the Ages & Stages Questionnaire© (ASQ-3) (Adjusted odds ratio (aOR) 1.44, 95%CI (1.02; −2.04)) [13]. In comparison to the pre-pandemic period, the COVID-19 pandemic has been associated with lower gross motricity (mean difference − 5.63, 95%CI (−8.75; −2.51)), fine motricity (mean difference −6.61, 95%CI (−10.00; −3.21)), and personal–social domain (mean difference −3.71, 95%CI (−6.61; −0.82)) in children at 6 months, using the ASQ-3 [16]. Furthermore, in utero exposure to high levels of stress, as seen during the January 1998 ice storm crisis in Quebec, has been associated with lower cognitive and language abilities in children when compared to those exposed to low or moderate levels of stress in utero [17].

Hence, the impact of in utero exposure to maternal mental health during the critical period of the COVID-19 pandemic on children’s cognitive development needs to be investigated. Therefore, we aimed to quantify the association between in utero exposure to maternal depression, anxiety, stress, and the children’s cognitive development at 18 months of age, in a population-based cohort of children born during the COVID-19 pandemic, within the CONCEPTION Study.

## 2. Materials and Methods

### 2.1. Data Source

This study used data from the CONCEPTION Study, which has been extensively described [10,11,18,19]. In brief, the CONCEPTION Study is a prospective mother–child cohort initiated shortly after the onset of the COVID-19 pandemic. It encompasses a population-based cohort of pregnant persons and their offspring born during the pandemic. Recruitment for this study started on 26 June 2020, and continued until 13 July 2022. A variety of recruitment methods were employed to ensure a representative cohort of pregnant persons during the COVID-19 pandemic. These methods included written press releases, commentaries, social media outreach, in-person engagement with community associations for new immigrants, and the use of QR codes in local obstetrics and gynecological clinics. Participants provided individual consent online, and data were collected through questionnaires on a secure platform using Survey Monkey®. Eligible participants for the CONCEPTION Study were individuals who were pregnant and were 18 years of age or older. They completed a baseline questionnaire (Q1) at the time of recruitment, which gathered information on sociodemographic characteristics, estimated delivery dates, comorbid health conditions during pregnancy, their treatments, over-the-counter medication use, maternal mental health during pregnancy, experiences with COVID-19 infection, and vaccination status. Subsequently, participants completed another questionnaire (Q2) two months after giving birth. This questionnaire gathered data on delivery experiences, including pregnancy outcomes (live birth, abortion, miscarriage, stillbirth), gestational age at delivery, maternal health outcomes (gestational diabetes, hypertension, preeclampsia, bleeding), mode of delivery, maternal mental health, experiences with COVID-19 since the last questionnaire, and characteristics of the offspring (birth weight, neonatal/pediatric ICU admissions, and prenatal outcomes). Lastly, participants filled out a questionnaire (Q3) at 18 months postpartum, which collected information about the children’s health characteristics at 18 months of age, including head circumference, medication use, hospitalization history, COVID-19 infections since birth, and cognitive developmental outcomes.

### 2.2. Study Population

The present study includes children born to participants who: (i) were pregnant and at least 18 years of age at the time of recruitment (completion of Q1 from June 2020 to December 2021), (ii) speak and understand English or French, and (iii) completed the Q3. The study period began at childbirth and ended at 18 months postpartum when the mothers completed a Q3 (from April 2022 to March 2023). The index date is defined as the child’s date of birth. 

### 2.3. Assessment of In Utero Exposure to Maternal Mental Health

Maternal mental health was assessed during pregnancy at the time of recruitment, using validated tools in both French and English for depression and anxiety. Depression during pregnancy was evaluated using the Edinburgh Postnatal Depression Scale (EPDS), the most widely used tool for assessing depression [20], sensitivity 86%, specificity 78% [21]. This scale reflects the mother’s feelings over the past seven days at the time of completion, through ten items (e.g., I have been able to laugh and see the funny side of things). Responses are scored from 0 to 3, based on the increasing severity of symptoms (e.g., 0 As much as I always could; 1 Not quite so much now; 2 Definitely not so much now; 3 Not at all). The sum of all items provides the EPDS total score from 0 to 30. Maternal symptoms of depression were categorized as follows: none to mild (scores < 9) and moderate to severe (scores ≥ 9) [21,22]. Anxiety during pregnancy was assessed using the validated self-reported General Anxiety Disorder-7 (GAD-7) questionnaire, sensitivity 83%, specificity 84% [23]. This screening tool measures GAD symptoms over the last two weeks at the time of completing the initial questionnaire, following DSM-IV guidelines. It consists of seven items (e.g., feeling nervous, anxious or on edge) scored from 0 to 3 (0 Not at all; 1 Several days; 2 More than half the days; 3 Nearly every day), with a score ranging from 0 to 21. We categorized maternal anxiety as follows: none to mild (scores ≤ 9) and moderate to severe (scores > 9) [24]. Moreover, we measured overall maternal stress related to COVID-19 using a visual analog scale, from the Coronavirus Perinatal Experiences Impact Survey (COPE-IS): ‘What has been your overall level of stress related to COVID-19?’ with answer options from 0 (indicating no stress) to 10 (indicating maximum stress). Finally, the use of antidepressants was assessed by inquiring whether participants had received a diagnosis of depression/anxiety (if yes, they were asked to specify the year) and whether they had used antidepressants in their lifetime. If a participant had received a diagnosis before the year of delivery and had used an antidepressant, they were considered exposed. 

### 2.4. Assessment of Children’s Cognitive Development

We assessed children’s cognitive development at 18 months using the validated tool ASQ-3 [25,26]. The ASQ-3 is a screening tool reported by parents, which assesses the children’s cognitive development across five domains: communication, gross motor, fine motor, problem solving, and personal–social. Each domain consists of six items, with a maximum possible score of 60. ASQ-3 is commonly used to identify the risk of developmental delay in children aged 2 to 66 months, with questionnaires available for each age group. In our study, we used the 18-month questionnaire for ASQ-3, which can be administered from 17 months 0 days to 18 months 30 days. Mothers were instructed to attempt the activities mentioned in the questionnaire with their child (e.g., does your child bend over or squat to pick up an object from the floor and then stand up again without any support?), preferably when the child was cooperative, well-rested, and fed. For each question, mothers were required to choose from three possible responses: “yes” (score = 10), “sometimes” (score = 5), or “not yet” (score = 0). If the child could perform the activity but refused to do so while filling the questionnaire, mothers were instructed to mark ‘Yes’ for that item. Additionally, the score obtained for each domain was adjusted using the ASQ-3 online calculator [27], when there were missing responses for one or two items at most in a domain. Participants who did not respond to three items or more of the assessment tool ASQ-3, in each domain, were excluded from the statistical analysis. Based on the ASQ-3 cutoffs, we categorized each domain into three groups: development on schedule (DS) (scores above the cut-off), requiring learning activities and monitoring (RLAM) (scores close to the cut-off), further assessment required with a professional (FARP) (scores below the cut-off) [28,29]. Communication scores were categorized as: DS (30–60); RLAM (15–29); FARP (0–14) [28,29]. Gross motor scores were categorized as: DS (46–60); RLAM (36–45); FARP (0–35). Fine motor scores were categorized as: DS (45–60); RLAM (35–44); FARP (0–34) [28,29]. Problem-solving scores were categorized as: DS (36–60); RLAM (26–35); FARP (0–25) [28,29]. Personal–social scores were categorized as: DS (38–60); RLAM (27–37); FARP (0–26) [28,29].

### 2.5. Covariates

Several covariates were considered as confounders in our adjusted models. First, we considered known maternal risk factors associated with our exposure of interest (maternal mental health—depression, anxiety and stress), namely: (1) maternal sociodemographic characteristics including maternal age at recruitment [30], ethnicity (Caucasian/white, other) [31], marital status (living alone or not) [30], living area (urban, suburban or rural), annual household income in Canadian dollars (≤$90,000, $90,001–120,000, $120,001–$150,000, ≥$150,001) [32], pre-pregnancy height and weight to calculate body mass index (BMI) [33], years of education (continuous); (2) maternal comorbidities before and during pregnancy, including hypertension [34], diabetes [35], and asthma [34,35]; (3) medication use during pregnancy; [36,37] and, (4) the year of recruitment of the mothers and gestational age at recruitment were considered, since maternal prenatal mental health was associated with the timing of the pandemic wave and trimester of gestation [11]. In addition, we measured the objective stress and hardship experienced by the mother using an instrument created for the CONCEPTION Study: the CONCEPTION study assessment of stress from COVID-19—150 points (CASC150). The CASC150 is comprised of three subscales: the threat faced due to the pandemic, level of financial loss, and the change in the daily life and pregnancy plans experienced due to the COVID-19 crisis.

### 2.6. Statistical Analysis

First, descriptive analyses were conducted to summarize children’s characteristics including sex, age, gestational age at birth, prematurity (<37 weeks gestation), birth weight (grams), low birth weight (<2500 g), delivery mode, jaundice, malformation or physical anomalies, COVID-19 diagnosis, and ASQ-3 domain scores, according to exposure to depression and anxiety in utero.

We then described maternal characteristics, including maternal mental health scores (i.e., EPDS, GAD-7, and stress), antidepressant use during pregnancy, CASC-150 scores, annual household income, marital status, living area, pre-pregnancy BMI, alcohol consumption, tobacco and cannabis smoking, education years, hypertension before/during pregnancy, diabetes before/during pregnancy, history of asthma, and over-the-counter medication use during pregnancy according to exposure to depression and anxiety in utero. The unit of analysis was one child. Pearson’s Chi-squared test or Fisher’s exact test (sample ˂ 5) were performed for categorical variables, and for continuous variables, the t-test was performed. Multinomial logistic regression models were constructed to assess the association between in utero exposure to maternal depression, anxiety, stress, antidepressants, and ASQ-3 categories in five domains: communication, gross motor, fine motor, problem solving, and personal–social development. These models were adjusted for potential confounding variables associated with prenatal mental health and child cognitive development identified in the literature (listed above), as well as CASC-150 scores. We estimated crude OR as well as aOR and 95% CI to measure these associations. Using multinomial logistic regressions allows us to extend binary logistic regression to handle scenarios where there are more than two categories in the dependent variable. Given the presence of missing data, multiple imputation was performed on the potential covariates listed above to perform multivariate multinomial logistic regression. When considering maternal mental health as the in utero exposure, we adjusted for all other maternal mental health variables that were measured. For example, if in utero exposure to depression was considered as the exposure of interest, we included in utero exposure to anxiety, stress and antidepressant in the model.

### 2.7. Sensitivity Analyses

We performed sensitivity analyses within a sub-cohort restricted to children aged 17 to 19 months, to determine if there is a difference with the main results. We performed sensitivity analyses to assess whether COVID-19 infection in children and the sex of the child act as effect modifiers, by conducting a logistic regression for each ASQ-3 domain categories, stratified on the children’s COVID-19 infection status and the children’s sex. Crude ORs and aORs with 95% CI were calculated to determine this association, using logistic regression models. Potential confounders were included based on the variance inflation factor to detect multicollinearity, for models that did not converge. For these sensitivity analyses, scores of ASQ-3 domains were defined dichotomously: DS, RLAM/ FARP. Communication scores were categorized as: DS/RLAM (14–60); FARP (0–14) [28,29]. Gross motor: DS/RLAM (36–60); FARP (0–35) [28,29]. Fine motor: DS/ RLAM (35–60); FARP (0–34) [28,29]. Problem-solving scores: DS/RLAM (26–60); FARP (0–25) [28,29]. Personal and social: DS/RLAM (27–60); FARP (0–26) [28,29]. 

Statistical analyses were performed using RStudio (Version 4.3.1).

### 2.8. Ethics

The CHU Sainte-Justine’s Research Ethics Committee approved the study (no. MP-21–2021–2973).

## 3. Results

### 3.1. Description of Children and Their Mothers

**Figure 1:**
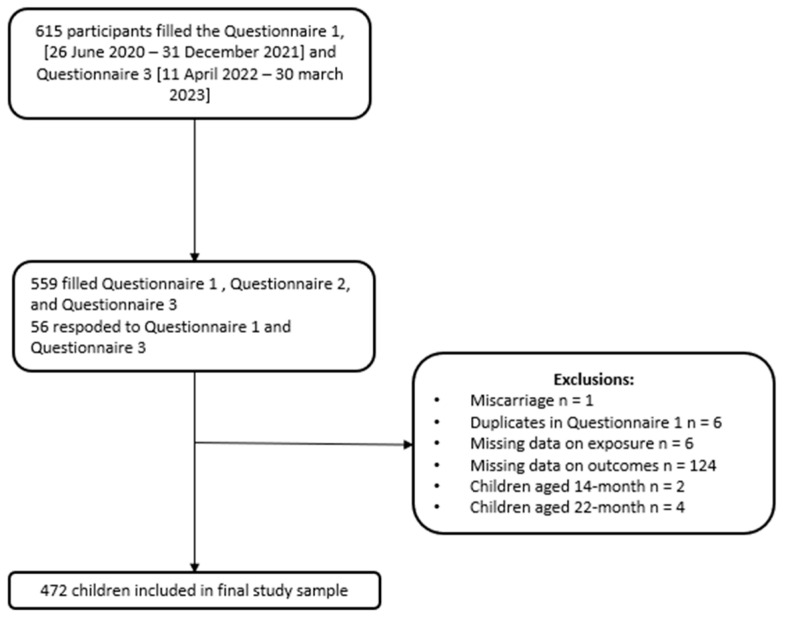
Flow chart of child cohort.

A total of 615 pregnant persons completed the questionnaire at 18 months postpartum. After excluding participants with miscarriage (n = 1), Q1 duplicates (n = 6), missing data for exposure (n = 6) or outcome (n = 124), and children aged <14 months (n = 2) or >22 months (n = 4), the final study sample consisted of 472 children (Figure 1). Among the 472 children included, 199 (42.2%) were exposed to symptoms of moderate to severe depression (EPDS ≥ 9) in utero, while 47 (10%) were exposed to moderate to severe symptoms of anxiety (GAD-7 > 9). Among children exposed to moderate to severe depression in utero, the prevalence of females was higher compared to those not exposed (54.0% vs. 44.2%, *p* = 0.049). The same trend was observed for medical conditions such as malformation and jaundice (10.6% vs. 5.3%, *p* = 0.041; 27.8% vs. 17.6%, *p* = 0.018). Gestational age at birth was lower among children exposed to moderate to severe symptoms of depression (39.3, SD 1.6 vs. 38.9, SD 1.8, *p* = 0.042). (Table 1).

Among mothers of children exposed to moderate to severe depression in utero, hypertension (15.2% vs. 7.4% *p =* 0.007), medication during pregnancy (76% vs. 63.7% *p* = 0.005), mean score of CAS150 (30.8, SD 10.7 vs. 25.2, SD 8.5 *p* < 0.001), history of anxiety (23.2% vs. 11% *p* < 0.001), antidepressant use before and/or during pregnancy (28.1% vs. 15.4% *p* < 0.001), were higher compared to mothers of children not exposed (Table 2). In addition, the mean gestational age at recruitment was higher in those exposed to moderate to severe depression in utero than those not exposed (20.9, SD 8.0 vs. 19.4, SD 8.2, *p =* 0.038). Regarding mothers of children exposed to moderate to severe anxiety in utero, results suggest significant association between maternal anxiety status and area of residence (*p* = 0.022). Diabetes (21.7% vs. 10.7% *p =* 0.027), asthma (19.5% vs. 8.8% *p =* 0.047), and medication use (82.2% vs. 67.5% *p =* 0.042) were higher among mothers of children exposed to moderate to severe anxiety in utero than among mothers of children exposed to no to mild anxiety. However, education years were higher among those exposed to no to mild anxiety than those exposed to moderate to severe anxiety (17.6 SD 4.4 vs. 15.2 SD 6.8). The mean score of CASC150 was significantly higher among mothers of children exposed to moderate to severe anxiety in utero, compared to those with no to mild anxiety exposure (33, SD 12.1 vs. 26.9, SD 9.4, *p =* 0.002). History of anxiety (34% vs. 14.2% *p* < 0.001) and antidepressant use (40.4% vs. 18.6% *p* < 0.001) were higher among mothers of children exposed to moderate to severe anxiety in utero than among mothers of children exposed to no to mild anxiety (Table 2).

### 3.2. Maternal Mental Health

The mean depression (EPDS) and anxiety (GAD-7) scores were of 7.9 (SD 5.4) and 4.3 (SD 3.9), respectively. Regarding the in utero exposure of stress related to the COVID-19 pandemic, the mean score was 4.5 (SD 2.1), and 20.8% of children were exposed to antidepressants (Table 2).

### 3.3. Children’s Cognitive Development Scores Using ASQ-3 Domains

Overall, the mean scores for each domain were as follows: communication 39.3 (SD 13.6), gross motor 53.5 (SD 11.0), fine motor 53.2 (SD 8.4), problem solving 45.3 (SD 10.9), personal and social 47.4 (SD 9.1) (Table 1).

Among children exposed to in utero moderate to severe depression, the mean score of problem solving 46.8 (SD 10.5) was significantly higher compared to those not exposed (44.3, SD 11.1, *p =* 0.014). In addition, there was a higher prevalence of DS for those exposed to moderate to severe depression in utero than those exposed to none to mild moderate to severe depression in utero (84.3% vs. 73.8%), and lower prevalence for RLAM (5.8% vs. 8.2%), and FARP (9.9% vs. 18.0%) *p =* 0.024 (Table 1).

In children exposed to moderate to severe anxiety in utero, the mean score of the communication domain was lower 32.8 (SD 15.2) compared to those exposed to no to mild anxiety 40 (SD 13.2). In addition, the prevalence of children with DS was lower for those exposed to moderate to severe anxiety in utero compared to those not exposed for communication (67.4% vs. 81.9%%, *p =* 0.009) and gross motricity (68.1% vs. 83.5% *p =* 0.013).

### 3.4. Association between Maternal Mental Health and Child Cognitive Development Using the Ages & Stages Questionnaire ASQ-3

After adjusting for potential confounders, communication and gross motricity domains were not associated with in utero exposure to moderate to severe depression, stress level related to the COVID-19 pandemic, and antidepressants. However, children exposed to moderate to severe anxiety in utero were associated with a higher risk of FARP (aOR 12.2, 95%CI (1.60;92.4)) for communication domain, and risk of RLAM for gross motricity (aOR 6.33, 95%CI (2.06;19.4)) compared to children not exposed (Table 3). In addition, exposure to moderate to severe depression in utero, moderate to severe anxiety, and stress level related to the COVID-19 pandemic were not associated with fine motricity. Moreover, the risk of RLAM was higher for children exposed to antidepressants in utero (aOR 4.11, 95%CI (1.00; 16)) compared to children not exposed. Regarding problem solving, no significant associations were reported with in utero exposure to moderate to severe anxiety, stress related to the COVID-19 pandemic, and antidepressants. Nevertheless, children exposed to moderate to severe depression in utero had lower risk of RLAM (aOR 0.48, 95%CI (0.24; 0.98)) than those exposed to none to mild depression in utero. As to personal–social, there was no significant association with in utero exposure to maternal mental health (moderate to severe depression, moderate to severe anxiety, stress related to the COVID-19 pandemic, and antidepressants) (Table 3).

### 3.5. Sensitivity Analyses

We assessed the relationship between prenatal maternal mental health and child cognitive development at 18 months of age using multivariate multinomial models including maternal mental health risk factors, restricted to children aged 17 to 19 months. After adjusting for potential confounders, communication, problem solving and personal–social were not associated with maternal mental health. However, children exposed to moderate to severe anxiety in utero are at higher risk for RLAM (aOR 5.53, 95%CI (1.52;20.1)) for gross motricity. In addition, children exposed to antidepressants were at higher risk for RLAM (aOR 4.70, 95%CI (1.15;19.1)) for fine motricity (Table SA1).

To assess if the child’s gender and COVID-19 infection are an effect modifier in the association between exposure to maternal mental health during pregnancy and children’s cognitive development, we performed sensitivity analyses stratified on these statuses. Among boys (n = 216), no significant associations were identified between any of the ASQ-3 domains and maternal mental health. Regarding girls (n = 202), the communication domain was not associated with exposure to moderate to severe depression in utero and stress related to the COVID-19 pandemic. However, girls exposed to moderate to severe depression in utero were associated with a lower risk of FARP (aOR 0.18, 95%CI (0.03; 0.73)) in gross motricity, and exposure to stress related to the COVID-19 pandemic was associated with a higher risk of FARP (aOR 1.44, 95%CI (1.04;2.06)) in gross motricity. In addition, fine motricity was not associated with in utero exposure to moderate to severe depression, stress related to the COVID-19 pandemic, and antidepressants. Regarding the problem-solving domain, girls exposed to moderate to severe anxiety in utero were associated with a higher risk of FARP (aOR 21.8, 95%CI (1.05;846)) compared to those not exposed. As for the personal–social domain, no significant associations were reported with in utero exposure to moderate to severe depression, moderate to severe anxiety, and stress related to the COVID-19 pandemic (Table SA2).

On one hand, among children infected by COVID-19, no significant associations were reported between communication, gross motricity, problem solving, personal–social domains and exposure to maternal mental health in utero. However, for fine motricity, in utero exposure to stress was associated with a lower risk of FARP for the child (aOR 0.38, 95%CI (0.10; 0.92)), among children infected by COVID-19. On the other hand, among children not infected by COVID-19, gross motricity, problem-solving, and personal–social were not associated with exposure to maternal mental health in utero. Nonetheless, for communication, children exposed to moderate to severe depression in utero were associated with a lower risk of FARP (aOR 0.05, 95%CI (0.00; 0.85)) and children exposed to moderate to severe anxiety in utero had a higher risk of FARP (aOR 86.0, 95%CI (3.25; 11,212)). As for fine motricity, exposure to moderate to severe depression in utero was associated with a lower risk of FARP (aOR 0.02, CI 95% (0.00; 0.38)), and a higher risk of FARP when exposed in utero to stress related to the COVID-19 pandemic (aOR 1.73, 95%CI (1.01;3.35)) (Table SA3).

## 4. Discussion

This study aimed to assess the exposure to prenatal maternal mental health (depression, anxiety, and overall pandemic-related stress) in utero, during the COVID-19 pandemic on children’s development at 18 months. We conducted this study using data from a large-scale Canadian cohort known as the CONCEPTION study, from which we evaluated the cognitive development of 472 children (Figure 1).

Our cohort consisted of children born during the COVID-19 pandemic, with an average age of 17.6 months (SD 1.25). After adjusting for potential confounders, communication and gross motricity domains were associated with a higher risk of FARP and RLAM for children exposed to moderate to severe anxiety in utero. In addition, fine motricity was associated with a higher risk of RLAM for children of mothers exposed to antidepressants before and/or during pregnancy. Regarding the problem-solving domain, it was associated with a lower risk of RLAM for those exposed to moderate to severe depression in utero; this could be attributed to the possibility that these mothers may be inclined to present their children in a positive way (Table 3). However, in the restricted cohort for children aged 17 to 19 months, gross motricity and fine motricity remained associated with in utero anxiety and antidepressant exposure (Table SA1). When stratifying on children’s sex, no significant association were reported among boys, but girls exposed to moderate to severe depression in utero seemed to have a lower risk of FARP for gross motricity domains (Table SA2). Moreover, exposure to stress related to COVID-19 in utero was associated with a higher risk of RLAM for gross motricity domains among girls. In addition, our results report a potential modifier effect for child infection status to COVID-19. Among children infected by COVID-19, exposure to stress related to the COVID-19 pandemic in utero was associated with a lower risk of FARP for the fine motor domain. Moreover, among children not infected with COVID-19, for the communication domain, exposure to moderate to severe depression in utero was associated with a lower risk of FARP, while exposure to moderate to severe anxiety in utero was associated with a higher risk of FARP. Regarding the fine motricity domain, exposure to moderate to severe depression in utero was associated with a lower risk of FARP, and stress level was associated with a higher risk of FARP (Table SA3).

Pre-pandemic studies studied the impact of maternal mental health on child cognitive development at different ages. Meanwhile, maternal mental health as well as the cognitive development of the child have been measured with various tools, which is important to consider while making comparisons. To the best of our knowledge, our study is the first to assess this association in the context of the COVID-19 pandemic.

Our results are in line with previous studies. Indeed, studies that used ASQ-3 reported no significant association (OR 1.03 95%CI (0.59;1.79)) [13] between problem solving (assessed by ASQ-3 for different ages) or cognitive development (β = 0.1 95%CI (−0.2; 0.3)) [38] and moderate to severe antenatal depression assessed using International Classification of Diseases, Ninth Revision, Clinical Modification codes or the Centers for Epidemiologic Studies Depression (CES-D) scale [13,38]. The studies found no significant association between the Spielberger State–Trait Anxiety Inventory (STAI) score and expressive language (β = −0.1 95%CI (−0.3; 0.1)), but a significant impact with overall cognition (β = −0.2 95%CI (−0.4; 0.0)) [38]. The Mother–Child Cohort in Crete, Greece (Rhea study) reported a negative association (β = —5.45, 95%CI (−10.44; −0.46)) between severe depression (EPDS ≥ 13) and the cognitive development scale (assessed with the Bayley Scales of Infant and Toddler Development—Third Edition (Bayley-III) at 18 months) [15]. In addition, this study reported that higher scores of the STAI score had a positive effect on expressive communication scores (β = 1.13, CI 95% (0.15, 2.11)) and no significant association with gross motor scores (β 0.05, 95%CI (−0.98; 1.07)) assessed with Bayley-III at 18 months [15]. The main difference is that the tool we used to assess cognitive development of the child (ASQ- 3) in our study is a screening tool filled in by parents, making it more susceptible to subjectivity, while the Bayley-III provides a more thorough and objective assessment of cognitive functions.

In addition, our results are in line with Zhang et al., who reported a significant association between maternal anxiety scores, using the Zhung self-rating Anxiety Scale (SAS) during pregnancy, and communication (β = −0.54, 95%CI (−0.92; −0.17)) as well as the gross motricity domain (β = −0.99, 95%CI (−1.63;−0.36)) at 6 and 12 months (ASQ-3), but only among boys. In addition, they reported a significant potential delay among girls for problem-solving skills (β = —2.05, 95%CI (−3.61; −0.50)) [39]. However, no significant association with depression during pregnancy was reported in boys, while a potential delay was observed in girls (β = —2.05, 95%CI (−3.61; −0.50)) [39].

Regarding exposure to stress, our results are not in line with previous findings about this association. Indeed, Project Ice Storm reported that maternal moderate–high stress had an impact on child’s communicative and language development during the Quebec Ice Storm in 1998 [17]. In addition, Karam et al. reported a significant positive association between the 4-item PSS and motor development assessed with Bayley-III [40]. However, our results are in line with the study of Keim et al., which reported no significant association between the 10-item Perceived Stress Scale (PSS) and cognitive development [38]. However, evidence assessing the use of antidepressants before/during pregnancy and their impact on child development is still unclear [37,41,42].

Regarding the child’s gender, our results suggest an effect modifier in the association between in utero exposure and maternal mental health. However, there is no clear evidence on the effect of child gender in this association. Indeed, the results of some studies suggest a poorer cognitive development in boys [43,44], while others suggest that this effect is more significant in girls [45]. Regarding COVID-19 infection of children, our results suggests that the status of infection is indeed an effect modifier in the association between in utero exposure to depression, anxiety, stress and antidepressants and cognitive outcomes at 18 months. However, there is a lack in literature about its impact on child’s cognitive development.

During the COVID-19 pandemic, one study suggested an association between poorer socio-cognitive skills, assessed using videotaped face-to-face interaction at 12 month, and prenatal stress due to the COVID-19 pandemic [46]. Conversely, a Serbian study that assessed prenatal anxiety using the STAI score reported a statistically significant association between the children’s socio-emotional status, using the scale for evaluation of psychophysiological abilities of children, and maternal trait anxiety (Pearson correlation coefficient *r* 0.184, *p* = 0.028) [47]. 

The mechanism through which maternal mental health during the prenatal period affects child cognitive development remains unclear. Two studies suggest that high levels of maternal cortisol in plasma and its ability to cross the placenta to reach the fetus may play a role in the impact of maternal mental health in utero and child cognitive development, due to down-regulation of placental 11β-hydroxysteroid dehydrogenase-2 [48,49]. 

### Strengths and Limitations

Our study is the first to assess the association between exposure to maternal mental health exposure in utero and child cognitive development using the ASQ-3, in the context of the COVID-19 pandemic. In addition, our study was conducted using data from a substantial population-based cohort known as the CONCEPTION Study. Data were collected prospectively at three different time points, ensuring a longitudinal follow-up that minimizes information bias. Notably, our study stands out as a cohort initiated early in the pandemic with a longitudinal follow-up design, capturing a comprehensive range of characteristics, particularly those related to maternal lifestyle and comorbidities. These rich data enabled us to make appropriate adjustments in multivariable models, thus minimizing the potential for confusion bias. To further mitigate selection bias, we employed diverse recruitment methods (e.g., social media, community centers) to assemble a representative cohort of pregnant persons in Canada within the unique context of the COVID-19 pandemic. In addition, we used validated tools available in both French and English to assess maternal depression, anxiety, and children’s cognitive development. The EPDS and GAD-7 scales, widely recognized and utilized in the field of prenatal mental health, were employed for assessing maternal depression and anxiety during pregnancy, respectively. Simultaneously, the ASQ-3 allowed us to evaluate multiple facets of a child’s development, encompassing communication, gross motricity, fine motricity, problem-solving, and personal–social domains. In addition, we used a fairly objective measure, the CASC150, to assess different aspects of maternal prenatal hardship during the COVID-19 pandemic.

However, we also acknowledge certain limitations. The questionnaires were lengthy to complete, taking 20 min for Q1, 15 min for Q2, and 20 min for Q3, which may have contributed to the observed missing values for some variables. However, we collected numerous variables of interest with a high completion rate (85% for baseline and the 2-month postpartum). In addition, we performed multiple imputations for the important adjustment variables. Our study is characterized by the fact that the participants had a higher annual income than the general Canadian population. In fact, the median household income in 2020 was $104,350 CAD, whereas 49.7% had a salary > $120,000 CAD in Canada (Statistics Canada, 2022) [50]. In addition, given that participation in the study was based on the participants’ willingness to take part, a selection bias could have preferentially resulted in individuals more concerned with the COVID-19 pandemic in the study. Lastly, with respect to outcomes, a potential source of bias is social desirability; mothers may have been positively biased in their responses about their children, possibly aligning their answers with social norms [51], leading to an underestimation of the association between maternal mental health and child cognitive development. However, multiple comparisons were performed for each exposure and each ASQ-3 domains. We may have found statistical significance by chance. However, thanks to the data collected in the CONCEPTION study, in our multinomial regression model we have adjusted for a good number of potential confounding factors.

## 5. Conclusions

In this population-based cohort study, our findings suggest that after adjusting for potential confounders, children born during the COVID-19 pandemic exposed to moderate to severe symptoms of anxiety, in utero, seemed to require further assessment by a professional for the communication domain and to require learning activities that improve gross motricity. In addition, those exposed, in utero, to antidepressants seemed to required learning activities and monitoring for fine motricity. However, children exposed to in utero symptoms of depression seemed to have a lower risk of required learning activities and monitoring for the problem-solving domain. Thus, it is crucial to continue monitoring children born during this health crisis to ensure an optimal environment for their learning and education leading to the children’s school entry. To continue these efforts, CONCEPTION is currently performing a follow-up study at 24 months to assess the cognitive development of children born during the COVID-19 pandemic using the diagnostic tool Bayley-III.

## Figures and Tables

**Table 1:** Characteristics of the child cohort according to exposure to depression and anxiety during in utero.

	Overall	Depression Status during Pregnancy ^h^			Anxiety Status during Pregnancy ^i^		
	N = 472 (%)	None to Mild, N = 273 (57.8%)	Moderate to Severe, N = 199 (42.2%)	*p*-Value	None to Mild, N = 425 (90%)	Moderate to Severe, N = 47 (10%)	*p*-Value
**Newborn sex**				**0.049**			0.79
Female	202 (48.3)	107 (44.2)	95 (54.0)		183 (48.5)	19 (46.3)	
Male	216 (51.7)	135 (55.8)	81 (46.0)		194 (51.5)	22 (53.7)	
Missing	54	31	23		48	6	
**Gestational age at birth, weeks, Mean (SD)**	39.1 (1.7)	39.3 (1.6)	38.9 (1.8)	**0.042**	39.2 (1.7)	38.7 (1.5)	0.064
**^a^** **Prematurity**	19 (4.4)	8 (3.2)	11 (6.1)	0.16	17 (4.4)	2 (4.7)	>0.99
Missing	45	26	19		41	4	
**Malformation**	32 (7.5)	13 (5.3)	19 (10.6)	**0.041**	26 (6.8)	6 (14.0)	0.12
Missing	46	27	19		42	4	
**Weight at birth, kilogram, mean (SD)**	3.3 (0.5)	3.3 (0.5)	3.3 (0.6)	0.48	3.3 (0.5)	3.2 (0.4)	0.15
Missing	56	31	25		49	7	
**^b^** **Low birth weigth**	25 (6.0)	12 (4.9)	13 (7.4)	0.29	22 (5.8)	3 (7.3)	0.72
Missing	52	29	23		46	6	
**Breastfeeding**	332 (78.5)	196 (80.7)	136 (75.6)	0.21	302 (79.5)	30 (69.8)	0.14
Missing	49	30	19		45	4	
**Jaundice**	83 (22.0)	38 (17.6)	45 (27.8)	**0.018**	73 (21.5)	10 (26.3)	0.49
Missing	94	57	37		85	9	
**Delivery mode**				0.27			0.072
Caesarean, planned	53 (13.3)	26 (11.4)	27 (15.9)		45 (12.6)	8 (19.5)	
Caesarean, urgent	51 (12.8)	33 (14.4)	18 (10.6)		50 (14.0)	1 (2.4)	
Vaginal	295 (73.9)	170 (74.2)	125 (73.5)		263 (73.5)	32 (78.0)	
**Missing**	73	44	29		67	32 (78.0)	
**Child COVID-19 infection**	264 (56.2)	158 (57.9)	106 (53.8)	0.38	243 (57.4)	21 (44.7)	0.094
Missing	2	0	2		2	0	
**Third edition of Ages & Stages questionnaire domains**							
**Communication score, mean (SD)**	39.3 (13.6)	39.1 (13.1)	39.5 (14.2)	0.78	40 (13.2)	32.8 (15.2)	**0.003**
Missing	1	0	1		0	1	
**^c^** **Communication categories**				0.70			**0.009**
DS	379 (80.5)	220 (80.6)	159 (80.3)		348 (81.9)	31 (67.4)	
RLAM	11 (2.3)	5 (1.8)	6 (3.0)		7 (1.6)	4 (8.7)	
FARP	81 (17.2)	48 (17.6)	33 (16.7)		70 (16.5)	11 (23.9)	
Missing	1	0	1		0	1	
**Gross motor score, mean (SD)**	53.5 (11.0)	53.4 (10.8)	53.6 (11.3)	0.88	53.8 (10.7)	50.7 (13.0)	0.13
Missing	2	2	0		2	0	
**^d^** **Gross motor categories**				0.85			**0.013**
DS	385 (81.9)	223 (82.3)	162 (81.4)		353 (83.5)	32 (68.1)	
RLAM	41 (8.7)	22 (8.1)	19 (9.5)		36 (8.5)	5 (10.6)	
FARP	44 (9.4)	26 (9.6)	18 (9.0)		34 (8.0)	10 (21.3)	
Missing	2	2	0		2	0	
**Fine motor score, mean (SD)**	53.2 (8.4)	52.8 (8.7)	53.7 (8.0)	0.28	53.4 (8.0)	51.3 (11.5)	0.22
Missing	2	2	0		2	0	
**^e^** **Fine motor categories**				0.81			0.19
DS	419 (89.1)	241 (88.9)	178 (89.4)		379 (89.6)	40 (85.1)	
RLAM	12 (2.6)	8 (3.0)	4 (2.0)		9 (2.1)	3 (6.4)	
FARP	39 (8.3)	22 (8.1)	17 (8.5)		35 (8.3)	4 (8.5)	
Missing	2	2	0		2	0	
**Problem-solving score, mean (SD)**	45.3 (10.9)	44.3 (11.1)	46.8 (10.5)	**0.014**	45.4 (10.9)	44.5 (11.4)	0.60
Missing	14	6	8		12	2	
**^f^** **Problem solving categories**				**0.024**			0.71
DS	358 (78.2)	197 (73.8)	161 (84.3)		322 (78.0)	36 (80.0)	
RLAM	33 (7.2)	22 (8.2)	11 (5.8)		29 (7.0)	4 (8.9)	
FARP	67 (14.6)	48 (18.0)	19 (9.9)		62 (15.0)	5 (11.1)	
Missing	14	6	8		12	2	
**Personal and social score, mean (SD)**	47.4 (9.1)	46.8 (9.4)	48.3 (8.5)	0.076	47.6 (9.0)	46 (9.5)	0.28
Missing	10	5	5		9	1	
**^g^** **Personal and social categories**				0.36			0.37
DS	406 (87.9)	234 (87.3)	172 (88.7)		368 (88.5)	38 (82.6)	
RLAM	13 (2.8)	10 (3.7)	3 (1.5)		11 (2.6)	2 (4.3)	
FARP	43 (9.3)	24 (9.0)	19 (9.8)		37 (8.9)	6 (13.0)	
Missing	10	5	5		9	1	

SD, standard deviation; DS, Development on schedule; RLAM, Required learning activities and monitoring; FARP, further assessment required by professional. ^a^ Preterm birth is defined as gestational age at delivery less than 37 weeks. ^b^ Low birth weight defined as birth weight at delivery less than 2500 g. ^c^ Communication scores categorized as follows: DS above the cut-off, the child appears to be on schedule (30–60); RLAM close to the cut-off, the child needs to be provided with learning activities (15–29); FARP below the cut-off, the child may need further assessment by a professional (0–14). ^d^ Gross motor scores categorized as follows: DS above the cut-off, the child appears to be on schedule (46–60); RLAM close to the cut-off, the child needs to be provided with learning activities (36–45); FARP below the cut-off, the child may need further assessment by a professional (0–35). ^e^ Fine motor scores categorized as follows: DS above the cut-off, the child appears to be on schedule (45–60); RLAM close to the cut-off, the child needs to be provided with learning activities (35–44); FARP below the cut-off, the child may need further assessment by a professional (0–34). ^f^ Problem-solving scores categorized as follows: DS above the cut-off, the child appears to be on schedule (36–60); RLAM close to the cut-off, the child needs to be provided with learning activities (26–35); FARP below the cut-off, the child may need further assessment by a professional (0–25). ^g^ Personal–social scores categorized as follows: DS above the cut-off, the child appears to be on schedule (38–60); RLAM close to the cut-off, the child needs to be provided with learning activities (27–37); FARP below the cut-off, the child may need further assessment by a professional (0–26). ^h^ Using the Edinburgh postnatal depression scale cut-off as follows: scale 0 to 8 means no to mild in utero exposure to maternal depression, scale 9 and more means in utero exposure to moderate to severe maternal depression. ^i^ Using the generalized anxiety disorder 7-item scale cut-off as follows: scale 0 to 9 means no to mild maternal anxiety, scale 10 and more means in utero exposure to moderate to severe maternal anxiety. Bold numbers indicate significant association (*p* < 0.05). *p*-values were calculated using the chi-square or Fisher test when categories were lower than 5 for categorical variables, while standardized mean differences were calculated for continuous variables.

**Table 2:** Maternal characteristics according to exposure to depression and anxiety during pregnancy.

	Overall	^k^ Depression Status during Pregnancy			^l^ Anxiety Status during Pregnancy		
	N = 472 (%)	None to Mild, N = 273 (57.8%)	Moderate to Severe, N = 199 (42.2%)	*p*-Value	None to Mild, N = 425 (90%)	Moderate to Severe, N = 47 (10%)	*p*-Value
**Maternal age at baseline, Mean (SD)**	32.6 (4.0)	32.3 (3.7)	33 (4.4)	0.065	32.6 (3.9)	32.2 (4.6)	0.54
**Period of recruitment**				0.73			0.091
2020	390 (82.6)	227 (83.2)	163 (81.9)		347 (81.6)	43 (91.5)	
2021	82 (17.4)	46 (16.8)	36 (18.1)		78 (18.4)	4 (8.5)	
**Annual household income, CAD**				0.85			0.80
≤$90,000	102 (22.7)	55 (21.3)	47 (24.6)		93 (23.0)	9 (20.5)	
$90,001–120,000	124 (27.6)	71 (27.5)	53 (27.7)		112 (27.7)	12 (27.3)	
$120,001–150,000	87 (19.4)	52 (20.2)	35 (18.3)		80 (19.8)	7 (15.9)	
≥$150,000	136 (30.3)	80 (31.0)	56 (29.3)		120 (29.6)	16 (36.4)	
Missing	23	15	8		20	3	
**Marital status—Living alone**				0.15			>0.99
No	455 (96.8)	267 (97.8)	188 (95.4)		410 (96.7)	45 (97.8)	
Yes	15 (3.2)	6 (2.2)	9 (4.6)		14 (3.3)	1 (2.2)	
Missing	2	0	2		1	1	
**Area of residence**				0.10			**0.022**
Rural	52 (11.1)	23 (8.5)	29 (14.6)		51 (12.1)	1 (2.1)	
Suburban	194 (41.4)	118 (43.5)	76 (38.4)		167 (39.6)	27 (57.4)	
Urban	223 (47.5)	130 (48.0)	93 (47.0)		204 (48.3)	19 (40.4)	
Missing	3	2	1		3	0	
**^a^** **Education, years, mean (SD)**	17.3 (4.8)	17.6 (4.3)	17 (5.3)	0.22	17.6 (4.4)	15.2 (6.8)	**0.025**
Missing	2	0	2		2	0	
**Pre-pregnancy body mass index, Mean (SD)**	25.2 (5.3)	24.8 (4.9)	25.7 (5.7)	0.086	25 (5.3)	26.5 (5.3)	0.077
Missing	4	2	2		3	1	
**Ethnicity/race**							
Caucasian/white	439 (93.2)	256 (94.1)	183 (92.0)	0.36	397 (93.6)	42 (89.4)	0.35
Other	32 (6.8)	16 (5.9)	16 (8.0)		27 (6.4)	5 (10.6)	
Missing	1	1	0		1	0	
**Alcohol consumption**	10 (2.1)	6 (2.2)	4 (2.0)	>0.99	9 (2.1)	1 (2.1)	>0.99
Missing	3	2	1		3	0	
**Smoking**	4 (0.8)	3 (1.1)	1 (0.5)	0.64	3 (0.7)	1 (2.1)	0.34
Missing	1	1	0		1	0	
**Use of cannabis products**	3 (0.6)	1 (0.4)	2 (1.0)	0.58	3 (0.7)	0 (0.0)	>0.99
Missing	1	1	0		1	0	
**Diabetes**	55 (11.8)	31 (11.5)	24 (12.2)	0.82	45 (10.7)	10 (21.7)	**0.027**
Missing	5	3	2		4	1	
**Hypertension**	50 (10.7)	20 (7.4)	30 (15.2)	**0.007**	43 (10.2)	7 (15.2)	0.31
Missing	5	3	2		4	1	
**Asthma**	43 (9.8)	23 (9.2)	20 (10.8)	0.58	35 (8.8)	8 (19.5)	**0.047**
Missing	35	22	13		29	6	
**Tylenol use**	204 (44.3)	110 (41.4)	94 (48.5)	0.13	180 (43.4)	24 (53.3)	0.20
Missing	12	7	5		10	2	
**Over-the-counter medication**	129 (28.0)	67 (25.2)	62 (32.0)	0.11	113 (27.2)	16 (35.6)	0.24
Missing	12	7	5		10	2	
**^b^** **Medication use during pregnancy**	321 (68.9)	172 (63.7)	149 (76.0)	**0.005**	284 (67.5)	37 (82.2)	**0.042**
Missing	6	3	3		4	2	
**^c^ CASC150 score, mean (SD)**	27.5 (9.9)	25.2 (8.5)	30.8 (10.7)	**<0.001**	26.9 (9.4)	33 (12.1)	**0.002**
Missing	21	13	8		20	1	
**^d^ Threat score, mean (SD)**	3.4 (3.9)	2.9 (3.5)	4.1 (4.3)	**0.002**	3.2 (3.7)	4.8 (5.1)	**0.039**
Missing	2	1	1		2	0	
**^e^ Loss score, mean (SD)**	4.3 (6.9)	3.5 (5.8)	5.5 (8.0)	**0.003**	4 (6.7)	7 (7.9)	**0.017**
Missing	3	2	1		3	0	
**^f^ Change score, mean (SD)**	19.7 (4.5)	18.6 (4.1)	21.3 (4.6)	**<0.001**	19.5 (4.3)	21.7 (5.5)	**0.011**
Missing	16	10	6		15	1	
**History of depression**	67 (14.2)	33 (12.1)	34 (17.1)	0.12	61 (14.4)	6 (12.8)	0.77
**History of anxiety**	76 (16.2)	30 (11.0)	46 (23.2)	**<0.001**	60 (14.2)	16 (34.0)	**<0.001**
Missing	2	1	1		2	0	
**^g^** **Antidepressant use**	98 (20.8)	42 (15.4)	56 (28.1)	**<0.001**	79 (18.6)	19 (40.4)	**<0.001**
**Gestational age at recruitment, weeks, mean (SD)**	20 (8.2)	19.4 (8.2)	20.9 (8.0)	**0.038**	20.1 (8.2)	19.3 (8.2)	0.52
**^h^** **Maternal depression ^h^, mean (SD)**	7.9 (5.4)	4.1 (2.7)	13 (3.5)	**<0.001**	7 (4.8)	15.4 (4.8)	**<0.001**
**^i^ Maternal anxiety ^i^** **, mean (SD)**	4.3 (3.9)	2.4 (2.4)	7 (4.0)	**<0.001**	3.3 (2.5)	13.3 (2.8)	**<0.001**
**^j^ Maternal stress ^j^, mean (SD)**	4.5 (2.1)	3.6 (1.9)	5.7 (1.8)	**<0.001**	4.3 (2.0)	6.6 (1.9)	**<0.001**

CAD, Canadian dollars; SD, standard deviation. ^a^ Education years since the age of 6. ^b^ Medication use during pregnancy: included treatment for chronic diseases and for comorbidities. ^c^ CASC150 (CONCEPTION study Assessment of Stress from COVID-19—150 points) is an instrument measuring the overall objective hardship, or objective stress, experienced by the questionnaire respondent. It has a possible maximum score of 150 points. ^d^ Threat50 assesses the level of threat that the respondent faced due to COVID-19. It is measured by items indicating if the health or life of the respondent, or their close ones, was threatened due to COVID-19, e.g., if the respondent or people around them suffered from COVID-19 symptoms and their severity, as well as access to food and medical care. ^e^ Loss50 assesses the level of financial loss due to COVID-19. It is measured by items indicating if the respondent suffered from a loss of income or savings, job security, insurance, or investments, and how this loss of income affected their life (ability to afford childcare or mortgage). ^f^ Change50 assesses the amount of change in the daily life and pregnancy plans that the respondent experienced due to the COVID-19 crisis. It is measured by items indicating changes in daily and work routine, social distancing, pregnancy care support, pregnancy class and care practice, and birth plans, due to COVID-19. ^g^ Antidepressant use defined by diagnosis of depression or anxiety combined to the use of antidepressants before delivery. ^h^ Using the Edinburgh postnatal depression scale, continuously. ^i^ Using the generalized anxiety disorder 7-item scale, continuously. ^j^ Using the overall maternal stress related to COVID-19 measured on a scale from 0 (no stress) to 10 (extreme stress). ^k^ Using the Edinburgh postnatal depression scale cut-off as follows: scale 0 to 8 means no in utero exposure to maternal depression, scale 9 and more means in utero exposure to moderate to severe maternal depression. ^l^ Using the generalized anxiety disorder 7-item scale cut-off as follows: scale 0 to 9 means no maternal anxiety, scale 10 and more means in utero exposure to moderate to severe maternal anxiety. Bold numbers indicate significant association (*p <* 0.05). *p*-values were calculated using chi-square or Fisher test when categories were lower than 5 for categorical variables, while standardized mean differences were calculated for continuous variables.

**Table 3:** Association between in utero exposure to depression, anxiety, stress, antidepressants, and domains of the Ages & Stages questionnaire—third edition (ASQ-3).

	^e^ Development on Schedule	^f^ Activities, Monitoring Required	Crude OR (95%CI)	* Adjusted OR (95%CI)	^g^ Further Assessement Required	Crude OR (95%CI)	* Adjusted OR (95% CI)
**Communication**							
**^a^** **In utero** **exposure to maternal depression**							
None to mild (EPDS < 9)	220 (58.0)	48 (59.3)	Reference	Reference	5 (45.5)	Reference	Reference
Moderate to severe (EPDS ≥ 9)	159 (42.0)	33 (40.7)	0.95 (0.58; 1.55)	0.82 (0.43; 1.54)	6 (54.5)	1.66 (0.50; 5.54)	0.73 (0.13; 4.14)
**^b^** **In utero** **exposure to maternal anxiety**							
None to mild (GAD-7 ≤ 9)	348 (91.8)	70 (86.4)	Reference	Reference	7 (63.6)	Reference	Reference
Moderate to severe (GAD-7 > 9)	31 (8.2)	11 (13.6)	1.76 (0.85; 3.68)	2.32 (0.92; 5.86)	4 (36.4)	**6.41 (1.78; 23.1)**	**12.2 (1.60; 92.4)**
**^c^** **In utero** **exposure to stress related to the COVID-19 pandemic, mean (SD)**	4.5 (2.1)	4.5 (2.1)	1.00 (0.89; 1.12)	1.00 (0.87; 1.16)	5.1 (2.5)	1.15 (0.86; 1.54)	1.21 (0.80; 1.84)
**^d^** **In utero** **exposure to maternal antidepressants**							
No	307 (81.0)	58 (71.6)	Reference	Reference	9 (81.8)	Reference	Reference
Yes	72 (19.0)	23 (28.4)	1.69 (0.98; 2.92)	1.28 (0.48; 3.42)	2 (18.2)	0.95 (0.20; 4.48)	0.33 (0.02; 6.06)
**Gross motor**							
**In utero** **exposure to maternal depression**							
None to mild (EPDS < 9)	223.0 (57.9)	26.0 (59.1)	Reference	Reference	22.0 (53.7)	Reference	Reference
Moderate to severe (EPDS ≥ 9)	162.0 (42.1)	18.0 (40.9)	0.95 (0.51; 1.80)	0.64 (0.26; 1.55)	19.0 (46.3)	1.19 (0.62; 2.27)	0.89 (0.40; 2.00)
**In utero** **exposure to maternal anxiety**							
None to mild (GAD-7 ≤ 9)	353.0 (91.7)	34.0 (77.3)	Reference	Reference	36.0 (87.8)	Reference	Reference
Moderate to severe (GAD-7 > 9)	32.0 (8.3)	10.0 (22.7)	**3.24 (1.47; 7.17)**	**6.33 (2.06; 19.4)**	5.0 (12.2)	1.53 (0.56; 4.18)	1.33 (0.39; 4.57)
**In utero** **exposure to stress related to the COVID-19 pandemic, mean (SD)**	4.5 (2.1)	4.4 (1.8)	0.99 (0.85; 1.15)	0.96 (0.79; 1.17)	4.9 (2.1)	1.09 (0.94; 1.28)	1.16 (0.95; 1.41)
**In utero** **exposure to maternal antidepressants**							
No	310.0 (80.5)	32.0 (72.7)	Reference	Reference	32.0 (78.0)	Reference	Reference
Yes	75.0 (19.5)	12.0 (27.3)	1.55 (0.76; 3.15)	2.27 (0.59; 8.82)	9.0 (22.0)	1.16 (0.53; 2.54)	1.59 (0.42; 5.97)
**Fine motor**							
**In utero** **exposure to maternal depression**							
None to mild (EPDS < 9)	241.0 (57.5)	22.0 (56.4)	Reference	Reference	8.0 (66.7)	Reference	Reference
Moderate to severe (EPDS ≥ 9)	178.0 (42.5)	17.0 (43.6)	1.05 (0.54; 2.03)	1.55 (0.65; 3.69)	4.0 (33.3)	0.68 (0.20; 2.28)	0.25 (0.04; 1.70)
**In utero exposure to maternal anxiety**							
None to mild (GAD-7 ≤ 9)	379.0 (90.5)	35.0 (89.7)	Reference	Reference	9.0 (75.0)	Reference	Reference
Moderate to severe (GAD-7 > 9)	40.0 (9.5)	4.0 (10.3)	1.08 (0.37; 3.20)	1.18 (0.31; 4.50)	3.0 (25.0)	3.16 (0.82; 12.1)	7.73 (0.85; 70.0)
**In utero exposure to stress related to the COVID-19 pandemic, mean (SD)**	4.5 (2.1)	4.0 (2.2)	0.88 (0.75; 1.03)	0.85 (0.68; 1.05)	4.8 (2.3)	1.05 (0.80; 1.38)	1.07 (0.74; 1.53)
**In utero exposure to maternal antidepressants**							
No	334.0 (79.7)	30.0 (76.9)	Reference	Reference	10.0 (83.3)	Reference	Reference
Yes	85.0 (20.3)	9.0 (23.1)	1.18 (0.54; 2.58)	**4.11 (1.00; 16.9)**	2.0 (16.7)	0.79 (0.17; 3.65)	0.42 (0.02; 8.58)
**Problem solving**							
**In utero exposure to maternal depression**							
None to mild (EPDS < 9)	197 (55.0)	48 (71.6)		Reference	22 (66.7)		Reference
Moderate to severe (EPDS ≥ 9)	161 (45.0)	19 (28.4)	**0.48 (0.27; 0.86)**	**0.48 (0.24; 0.98)**	11 (33.3)	0.61 (0.29; 1.30)	0.48 (0.18; 1.26)
**In utero exposure to maternal anxiety**							
None to mild (GAD-7 ≤ 9)	322 (89.9)	62 (92.5)	Reference	Reference	29 (87.9)	Reference	Reference
Moderate to severe (GAD-7 > 9)	36 (10.1)	5 (7.5)	0.72 (0.27; 1.91)	0.95 (0.30; 3.01)	4 (12.1)	1.23 (0.41; 3.71)	1.34 (0.34; 5.32)
**In utero exposure to stress related to the COVID-19 pandemic, mean (SD)**	4.5 (2.1)	4.1 (1.9)	0.91 (0.80; 1.04)	1.03 (0.88; 1.20)	4.5 (2.3)	0.98 (0.83; 1.16)	1.05 (0.85; 1.29)
**In utero exposure to maternal antidepressants**							
No	281 (78.5)	56 (83.6)	Reference	Reference	27 (81.8)	Reference	Reference
Yes	77 (21.5)	11 (16.4)	0.72 (0.36; 1.43)	0.85 (0.24; 2.97)	6 (18.2)	0.81 (0.32; 2.03)	0.67 (0.12; 3.87)
**Personal–social**							
**In utero exposure to maternal depression in utero**							
None to mild (EPDS < 9)	234 (57.6)	24 (55.8)	Reference	Reference	10 (76.9)	Reference	Reference
Moderate to severe (EPDS ≥ 9)	172 (42.4)	19 (44.2)	1.08 (0.57; 2.03)	1.39 (0.62; 3.12)	3 (23.1)	0.41 (0.11; 1.51)	0.23 (0.04; 1.33)
**Anxiety in utero**							
None to mild (GAD-7 ≤ 9)	368 (90.6)	37 (86)	Reference	Reference	11 (84.6)	Reference	Reference
Moderate to severe (GAD-7 > 9)	38 (9.4)	6 (14)	1.57 (0.62; 3.96)	2.03 (0.68; 6.04)	2 (15.4)	1.76 (0.38; 8.24)	3.59 (0.45; 28.9)
**In utero exposure to stress related to the COVID-19 pandemic, mean (SD)**	4.5 (2.1)	4 (2.2)	0.89 (0.77; 1.04)	0.84 (0.69; 1.02)	4.7 (1.8)	1.04 (0.80; 1.36)	1.23 (0.86; 1.76)

SD, standard deviation. ^e^ ^a^ Using the Edinburgh postnatal depression scale cut-off as follows: scale 0 to 8 means no to mild in utero exposure to maternal depression, scale 9 and more means in utero exposure to moderate to severe maternal depression. ^b^ Using the generalized anxiety disorder 7-item scale cut-off as follows: scale 0 to 9 means no to mild maternal anxiety, scale 10 and more means in utero exposure to moderate to severe maternal anxiety. ^c^ Using the overall maternal stress related to COVID-19 measured on a scale from 0 (no stress) to 10 (extreme stress). ^d^ Antidepressant use defined by diagnosis of depression or anxiety combined to the use of antidepressants before delivery. ^e^ Development on schedule (DS) above the cut-off, the child appears to be on schedule. ^f^ Required learning activities and monitoring (RLAM) close to the cut-off, the child needs to be provided with learning activities. ^g^ Further assessment required by professional (FARP) below the cut-off. Communication scores categorized: Development on schedule (DS) above the cut-off, the child appears to be on schedule (30–60); Required learning activities and monitoring (RLAM) close to the cut-off, the child needs to be provided with learning activities (15–29); further assessment required by professional (FARP) below the cut-off, the child may need further assessment by a professional (0–14). Gross motor scores categorized: DS above the cut-off, the child appears to be on schedule (46–60); RLAM close to the cut-off, the child needs to be provided with learning activities (36–45); FARP below the cut-off, the child may need further assessment by a professional (0–35). Fine motor scores categorized: DS above the cut-off, the child appears to be on schedule (45–60); RLAM close to the cut-off, the child needs to be provided with learning activities (35–44); FARP below the cut-off, the child may need further assessment by a professional (0–34). Problem-solving scores categorized: DS above the cut-off, the child appears to be on schedule (36–60); RLAM close to the cut-off, the child needs to be provided with learning activities (26–35); FARP below the cut-off, the child may need further assessment by a professional (0–25). Personal and social scores categorized: DS above the cut-off, the child appears to be on schedule (38–60); RLAM close to the cut-off, the child needs to be provided with learning activities (27–37); FARP below the cut-off, the child may need further assessment by a professional (0–26). * Adjusted models for potential confounders: Communication: maternal depression, maternal age at recruitment, annual household income, pre-pregnancy body mass index, diabetes (including gestational diabetes), hypertension (including preeclampsia), education years, maternal anxiety, maternal stress related to the COVID-19 pandemic, period of recruitment, gestational age at delivery, CASC-150 score, medication use. Gross motor: maternal depression, maternal age at recruitment, annual household income, pre-pregnancy body mass index, diabetes (including gestational diabetes), hypertension (including preeclampsia), education years, maternal anxiety, maternal stress related to the COVID-19 pandemic, period of recruitment, gestational age at delivery, CASC-150 score, medication use, area of residence, maternal asthma, history of depression, ethnicity, history of anxiety. Fine motor: maternal depression, maternal age at recruitment, annual household income, pre-pregnancy body mass index, diabetes (including gestational diabetes), hypertension (including preeclampsia), education years, maternal anxiety, maternal stress related to the COVID-19 pandemic, period of recruitment, gestational age at delivery, CASC-150 score, medication use, area of residence, marital status, history of depression, history of anxiety, ethnicity. Problem-solving: maternal depression, maternal age at recruitment, annual household income, pre-pregnancy body mass index, diabetes (including gestational diabetes), hypertension (including preeclampsia), education years, maternal anxiety, maternal stress related to the COVID-19 pandemic, period of recruitment, gestational age at delivery, CASC-150 score, medication use, maternal asthma, history of depression, history of anxiety, ethnicity. Personal and social: maternal depression, maternal age at recruitment, annual household income, pre-pregnancy body mass index, diabetes (including gestational diabetes), hypertension (including preeclampsia), education years, maternal anxiety, maternal stress related to the COVID-19 pandemic, period of recruitment, gestational age at delivery, CASC-150 score, medication use, area of residence, marital status, maternal asthma, ethnicity, history of depression, history of anxiety. Bold numbers indicate significant association (*p* < 0.05).

## Data Availability

Anonymized individual-level data from the study including data dictionaries, data collection tools will be made available upon request. Requests for access will be reviewed by a data access committee.

## Supplementary Materials

The following supporting information can be downloaded at: https://www.mdpi.com/article/10.3390/jcm13041055/s1, Table SA1: Association between in utero exposure to depression, anxiety, stress, antidepressant use, and Ages & Stages questionnaire—third edition (ASQ-3)—cohort restricted to children aged 17 to 19 months; Table SA2: Association between in utero exposure to depression, anxiety, stress, antidepressant use, and Ages & Stages questionnaire—third edition (ASQ-3)—stratified by the child’s gender; Table SA3: Association between in utero exposure to depression, anxiety, stress, antidepressant use, and Ages & Stages questionnaire—third edition (ASQ-3)—stratified for child status regarding infection by COVID-19.
