# Supplementary material for: Maternal Mental Health in Pregnancy and Its Impact on Children’s Cognitive Development at 18 Months, during the COVID-19 Pandemic (CONCEPTION Study)
Source: J Clin Med. 2024 Feb 13;13(4):1055. doi: 10.3390/jcm13041055 (PMC10889100; doi:10.3390/jcm13041055)
Supplement: Supplementary file 1 [file jcm-13-01055-s001.zip › Questionnaires/Q1.pdf]

Welcome to the CONCEPTION Study

The arrival of COVID-19 and the World Health Organization's announcement of the pandemic status prompted many local and national governments to institute new guidelines on public health and hospital policies. These measures, aimed at limiting the spread of COVID-19 and diminishing the burden of the pandemic on the health system, may have an impact on the lives of pregnant women.

The CONCEPTION study aims to collect information on the impact of the public health and hospital policy guidelines related to the COVID-19 pandemic on the mental and physical health of pregnant women through an online questionnaire.

Let's find out if you are eligible to participate in the CONCEPTION study!

Assessment of eligibility

* Are you 18 years of age or older?

- ◯ Yes
- ◯ No

Assessment of eligibility

* Are you currently pregnant?

- ◯ Yes
- ◯ No, I delivered between March 13th and today
- ◯ No

CONCEPTION Study

Online Consent

CONCEPTION Study: Short- and long-term impact of COVID-19 public health guidelines and hospital policies on maternal and child mental and physical health.

Consent form
In order to make an informed decision regarding your participation in this research project, please read the content of this information and consent form carefully. If you decide to participate, please indicate your consent at the bottom of this page.

This project has been approved by the Research Ethics Committee of the Research Center of the Center hospitalier universitaire (CHU) Sainte-Justine (Institutional ethical review approval number: #**2021-2973**).

Please read this information and consent form carefully: PDF of informed and consent form (opens in new window)

I understand that the goal of Dr. Anick Bérard's research project is to collect and analyze responses to a short questionnaire on the impact of public and hospital health recommendations related to the COVID-19 pandemic on my mental and physical health. I had the opportunity to ask the research team questions and get answers if I chose to.

* I agree to participate in this research project, including the research data base, and I confirm to be 18 or older.

- ◯ Yes
- ◯ No

Please keep a copy of this page and of the consent and information form (save the document) for your files.

General and socio-demographic information

Let's begin your survey! This should only take you about 20 minutes.

When is your birthday?

	Day	Month	Year
Birth date			

How far along are you in your pregnancy? (in weeks)

When is your due date?

If you are unsure about your due date, please put the closest estimate.

Date

DD/MM/YYYY

Which professional follows you for your pregnancy? (Check all that apply)

- ☐ Family Physician
- ☐ Obstetrician/Gynaecologist
- ☐ Midwife
- ☐ Nurse Practitionner
- ☐ No follow-up

In which country do you live in?

If you live in Canada which province do you live in?

What is your height?

- ☐ cm
- ☐ ft/inch

If you are 5 feet and 4 inches, please write 5'4".

What was your weight **before** becoming pregnant?

☐ kg ☐ lbs

Weight

What is your **current** weight?

☐ kg ☐ lbs

Weight

Personal Experience with COVID-19

Have you **ever been** tested for COVID-19?

- ◯ Yes
- ◯ No

Personal Experience with COVID-19

If yes, **which type** of test did you receive? (Check all that applies)

- ☐ Nasal/Throat swab
- ☐ Blood test
- ☐ Symptoms and fever only

Have you tested **positive** on a COVID-19 test?

- ○ Yes
- ○ No

If yes, when was this?

Date your test was considered positive.

Date

DD/MM/YYYY

Personal Experience with COVID-19

If you believe that you have had COVID-19, how severe was it?

- ◯ None. I had no symptoms.
- ◯ Mild. Symptoms effectively managed at home.
- ◯ Moderate. Symptoms severe and required brief hospitalization.
- ◯ Severe. Symptoms severe and required ventilation (admission to intensive care and potential intubation).
- ◯ I do not believe that I had COVID-19.

If you believe that you have had COVID-19, have you recovered?

- ◯ Yes, totally
- ◯ Partially
- ◯ Not at all
- ◯ Unsure
- ◯ I do not believe that I had COVID-19

Personal Experience with COVID-19

Number of immediate family members diagnosed with COVID-19

Rate the symptoms of the person who was the most sick.

- No one in my immediate family was diagnosed with COVID-19.
- Mild. Symptoms were effectively managed at home.
- Moderate. Symptoms severed and required brief hospitalization.
- Severe. Symptoms severe and required ventilation (admission to the intensive care unit).

Number of extended family members and/or close friends diagnosed with COVID-19

Vaccination Acceptability

Did you take the flu vaccine during the 2020-2021 season (since November 2020)?

- ◯ Yes
- ◯ No

Please rate your knowledge of the severity of COVID-19 **in pregnancy?**

No Knowledge				Excellent Knowledge
◯	◯	◯	◯	◯

Please rate your knowledge of the COVID-19 vaccine(s) **in general?**

No Knowledge				Excellent Knowledge
◯	◯	◯	◯	◯

Please rate your knowledge of the COVID-19 vaccine(s) **in pregnancy?**

No Knowledge				Excellent Knowledge
◯	◯	◯	◯	◯

If it were available to you, would you accept the COVID-19 vaccine during your pregnancy?

- ◯ Yes
- ◯ No

Personal Experience with COVID-19 vaccines

If no or unsure, what are the reasons? (check all that apply)

- ☐ Public health officials advised against it
- ☐ Family doctor advised against it
- ☐ Obstetrician advised against it
- ☐ Nurse practitioner advised against it
- ☐ Midwife or Doula advised against it
- ☐ Lack of efficacy data in pregnancy
- ☐ Lack of safety data in pregnancy
- ☐ Lack of information on vaccines in pregnancy
- ☐ The vaccine was created too quickly
- ☐ The vaccine was approved too quickly
- ☐ I will not be able to take a second dose because it’s not available
- ☐ COVID-19 is not that serious - I do not need vaccination
- ☐ I had lower exposure to COVID-19
- ☐ Other, please specify

Did you receive the COVID-19 vaccine?

- ○ Yes
- ○ No

Personal Experience with COVID-19 vaccines

Which vaccine did you receive?

- ◯ Pfizer/BioNTech
- ◯ Moderna
- ◯ Oxford University/AstraZeneca
- ◯ Johnson & Johnson
- ◯ I don’t know
- ◯ Other, please specify

How many doses have you received so far?

- ◯ One
- ◯ Two

Personal Experience with COVID-19 vaccines

When did you receive the COVID-19 vaccine?

Vaccination Date

Date

DD/MM/YYYY

If yes, did you experience any of these side effects? (check all that apply)

- [ ] Pain and swelling at the site of infection
- [ ] Fever
- [ ] Chills
- [ ] Tiredness
- [ ] Headache
- [ ] Allergic reaction
- [ ] Joint pain
- [ ] Nausea and/or vomiting
- [ ] Feeling unwell
- [ ] Swollen lymph nodes
- [ ] No side effects
- [ ] Other, please specify

Personal Experience with COVID-19 vaccines

When did you receive the first dose of the COVID-19 vaccine?

Vaccination Date - Dose 1

Date

DD/MM/YYYY

When did you receive the second dose of the COVID-19 vaccine?

Vaccination Date - Dose 2

Date

DD/MM/YYYY

If yes, did you experience any of these side effects? (check all that apply)

- ☐ Pain and swelling at the site of infection
- ☐ Fever
- ☐ Chills
- ☐ Tiredness
- ☐ Headache
- ☐ Allergic reaction
- ☐ Joint pain
- ☐ Nausea and/or vomiting
- ☐ Feeling unwell
- ☐ Swollen lymph nodes
- ☐ No side effects
- ☐ Other, please specify

Sociodemographic Information

How many years of schooling have you completed, as of the age of 6?

[ ]

What was your main occupation status in February of 2020?

- ◯ Student/intern
- ◯ Employed - full time
- ◯ Employed - part time
- ◯ Self-employed
- ◯ Unemployed
- ◯ On welfare
- ◯ Prefer not to answer

How do you see yourself?

- ◯ Caucasian/White
- ◯ Black
- ◯ Asian
- ◯ Hispanic
- ◯ Aboriginal (North American Indians, Métis or Inuit [Inuk])
- ◯ Other
- ◯ Prefer not to answer

Which of these statements best describes your living situation?

- ◯ Living alone or single mother
- ◯ Living with a partner/married
- ◯ Living with parents/family
- ◯ Other
- ◯ Prefer not to answer

Which of the following best describes the area you live in?

- ◯ Urban
- ◯ Suburban
- ◯ Rural

If you live in Canada, please indicate the first 3 caracters of your postal code.

[ ]

What was your household income before taxes in 2019? If you are married or living with a partner, please indicate the family income before taxes (in Canadian dollars).

- <$30,000
- $30,001-$60,000
- $60,001-$90,000
- $90,001-$120,000
- $120,001-$150,000
- $150,001-$180,000
- >$180,000
- Prefer not to answer

Pregnancy history

Is this the first time you are pregnant?

- ◯ Yes
- ◯ No

Which of the following best describes your pregnancy?

- ◯ Singleton (1 baby)
- ◯ Multiple (more than 3 babies)
- ◯ Twins (2 babies)

How many deliveries, abortions or miscarriages have you had before your present pregnancy?

	Number
Deliveries	
Abortions	
Miscarriages	

How many children do you have now?

Number of children	

Pregnancy experience related to the COVID-19 pandemic

How well are you currently being supported by your primary prenatal care provider(s)?

- ○ Very well supported
- ○ Moderately well supported
- ○ Not very well supported
- ○ No support at all

Has the support you receive from your primary prenatal care provider(s) changed due to the COVID-19 pandemic?

- ○ Significantly worsened
- ○ Somewhat worsened
- ○ No change
- ○ Somewhat improved
- ○ Significantly improved

What resources are currently available to you from your prenatal care practice? (Check all that apply)

- ☐ Regular in-person appointment
- ☐ Virtual care appointements (e.g. video calls, Zoom, Skype)
- ☐ Phone call appointments
- ☐ Online messaging portal for questions/concerns
- ☐ Emergency care
- ☐ Home blood pressure monitoring
- ☐ Home fetal heart rate monitoring
- ☐ Don't know
- ☐ Other

How concerned are you about possible **future hospital policy changes** during your baby's birth (delivery/postnatal stay) as a result of the COVID-19 pandemic?

No concern ○ ○ ○ ○ ○ ○ ○ Highly concerned

Pregnancy experiences related to the COVID-19 pandemic

Have you experienced a change in the **type of prenatal classes/information** as a result of the COVID-19 pandemic? (check all that apply).

- [ ] Not applicable: I was not receiving/planning to receive any prenatal education before COVID-19
- [ ] No change in my access to prenatal education
- [ ] My classes were cancelled completely
- [ ] My classes changed to online/virtual classes
- [ ] My classes were replaced by reading material only
- [ ] I went online to get prenatal education and information
- [ ] Other

Have you experienced a change in prenatal care in any of the following areas as result of the COVID-19 pandemic? (Check all that apply)

- [ ] Cancellations of prenatal visit(s)
- [ ] Reduction in frequency of perinatal visit(s)
- [ ] Rushed appointments
- [ ] Postponed appointment(s)
- [ ] Change of prenatal healthcare provider(s)
- [ ] Change in medications or treatment
- [ ] Prenatal visit(s) changed from in person to virtual
- [ ] Cancellation of hospital tours
- [ ] No change

Have you experienced a change in your diet since the beginning of the pandemic?

- ○ I have been eating much worse than before
- ○ I have been eating somewhat worse than before
- ○ No change in diet
- ○ I have been eating better than before
- ○ I have been eating much better than before

Before learning about the COVID-19 pandemic, where were you planning on giving birth?

- ○ Hospital
- ○ Birth center located in a hospital
- ○ Birthing/maternity house (**NOT** located in a hospital)
- ○ Home
- ○ I hadn't decided yet
- ○ Other

As of today, where were you planning on giving birth?

- ○ Hospital
- ○ Birth center located in a hospital
- ○ Birthing/maternity house (**NOT** located in a hospital)
- ○ Home
- ○ I haven't decided yet
- ○ Other

Changes in birth plan related to the COVID-19 pandemic

Considering the changes in your birth plan

Are you concerned that any of the following may happen to you during or following your labor and delivery as a result of the COVID-19 pandemic?

	Not at all concerned	A little concerned	Moderately concerned	Very concerned	Not applicable
Reduced access to preferred medications (i.e. nitrous oxide, epidural)	○	○	○	○	○
Not being able to have a water birth	○	○	○	○	○
My primary health care provider will be unavailable for home birth	○	○	○	○	○
My primary health care provider will be unavailable for hospital birth	○	○	○	○	○
My doula/midwife will not be permitted to attend the baby's delivery at the hospital	○	○	○	○	○
Support people (e.g. partner, family member) will not be permitted to attend baby's delivery	○	○	○	○	○
I may not have the support I need to have the more natural delivery I was planning for (e.g., bath, massage, walking, pressure points, ball, etc.)	○	○	○	○	○
I may be separated from baby after delivery	○	○	○	○	○
I may be sent home early after labor (i.e. shorter stay in hospital than planned)	○	○	○	○	○

	Not at all concerned	A little concerned	Moderately concerned	Very concerned	Not applicable
I may not be provided adequate opportunity for immediate skin-to skin contact with my newborn	○	○	○	○	○
I may not be provided adequate opportunity to try to initiate breastfeeding	○	○	○	○	○
Family and friends may not be able to visit me and my baby (e.g., due to social distancing or travel restrictions)	○	○	○	○	○
My baby may receive less optimal postnatal care due to the COVID-19 pandemic (e.g., fewer checkups after birth)	○	○	○	○	○
I may not have access to lactation support following discharge from the hospital	○	○	○	○	○

Concerns with COVID-19 related to your delivery

Are you concerned that any of the following may happen to you during or following your labor and delivery as a result of the COVID-19 pandemic?

	Not at all concerned	A little concerned	Moderately concerned	Very concerned	Not applicable
I may have birth complications due to contracting COVID-19	○	○	○	○	○
I may be exposed to COVID-19 during pregnancy	○	○	○	○	○
I may be exposed to COVID-19 during labor/delivery or shortly thereafter	○	○	○	○	○
My baby may become infected with COVID-19 after birth	○	○	○	○	○
I will be infected with COVID-19 and be unable to care for my child	○	○	○	○	○

Present experiences related to the COVID-19 pandemic

How has the COVID-19 pandemic changed your financial situation? (check all that apply)

- [ ] My situation has not changed
- [ ] Decreased take-home pay
- [ ] Increased take-home pay
- [ ] Loss of job
- [ ] Secured a job
- [ ] Decreased job security
- [ ] Loss of health insurance
- [ ] Increased job security
- [ ] Reduced ability to afford childcare
- [ ] Reduced ability to afford rent/mortgage
- [ ] Decrease in value of your retirement, investments or savings
- [ ] Moved to remote work or working from home
- [ ] Reduction in work hours
- [ ] Increased hours
- [ ] Increased responsibilities

Present experiences related to the COVID-19 pandemic

We want to know how much COVID-19 has changed the following areas of your life.

Family Income/Employment:

- ◯ No change.
- ◯ Mild. Small change. Able to meet all needs and pay bills.
- ◯ Moderate. Having to make cuts in spending but able to meet basic needs and pay bills.
- ◯ Severe. Unable to meet basic needs and/or pay bills.

Daily routine:

- ◯ No change.
- ◯ Mild. Change in only one area (e.g. work, education, social life, hobbies, religious activities).
- ◯ Moderate. Change in two areas (e.g. work, education, social life, hobbies, religious activities).
- ◯ Severe. Change in three or more areas (e.g. work, education, social life, hobbies, religious activities).

Food access:

- ◯ No change.
- ◯ Mild. Enough food but difficulty getting to stores and/or finding needed items.
- ◯ Moderate. Occasionally without enough food and/or good quality (e.g., healthy) foods.
- ◯ Severe. Frequently without enough food and/or good quality (e.g., healthy) foods.

Medical health care access, not including mental health:

- ◯ No change.
- ◯ Mild. Appointments moved to telehealth, meaning over the phone or by the internet.
- ◯ Moderate. Delays or cancellations in appointments and/or delays in getting prescriptions; but changes have minimal impact on health.
- ◯ Severe. Unable to access needed care resulting in moderate to severe impact on health.

Mental health treatment access:

- Not applicable.
- No change.
- Mild. Appointments moved to telehealth, meaning over the phone or by the internet.
- Moderate. Delays or cancellations in appointments and/or delays in getting prescriptions; but changes have minimal impact on health.
- Severe. Unable to access needed care resulting in moderate to severe impact on health.

Access to family, extended family and non-family social supports:

- No change.
- Mild. Continued visits with social distancing and/or regular phone calls and/or televideo or social media contacts.
- Moderate. Loss of in-person and remote contact with a few people, but not all supports are lost.
- Severe. Loss of in-person and remote contact with all supports.

Work situation:

- No change.
- I am unemployed.
- Moved to remote work or working for home.
- Reduction in work hours.
- Increased work hours.
- Increased responsibilities.

Satisfaction with Life

We are now going to talk about the quality of your life in relation to both your pregnancy and personal health.

In general, would you say your health is :

- ◯ Excellent
- ◯ Very good
- ◯ Good
- ◯ Fair
- ◯ Poor

Below are five statements that you may agree or disagree with. Using the 1 - 7 scale below, indicate your agreement with each item by placing the appropriate number on the line preceding that item. Please be open and honest in your responding.

	1-Strongly agree	2-Agree	3-Slightly agree	4-Neither agree nor disagree	5-Slightly disagree	6-Disagree	7-Strongly disagree
In most ways my life is close to my ideal.	◯	◯	◯	◯	◯	◯	◯
The conditions of my life are excellent.	◯	◯	◯	◯	◯	◯	◯
I am satisfied with my life.	◯	◯	◯	◯	◯	◯	◯
So far I have gotten the important things I want in life.	◯	◯	◯	◯	◯	◯	◯
If I could live my life over, I would change almost nothing.	◯	◯	◯	◯	◯	◯	◯

If you think about life **prior to the COVID-19 pandemic**, how would you rate your satisfaction with your life?

Very satisfied	Satisfied	Dissatisfied	Very dissatisfied
◯	◯	◯	◯

If you think about **life today**, how would you rate your satisfaction with your life?

Very satisfied	Satisfied	Dissatisfied	Very dissatisfied
◯	◯	◯	◯

Stress related to COVID-19 pandemic

Stress and discord in the family or with other people you live with:

- No change.
- Mild. Family members occasionally short-tempered with one another; no physical violence.
- Moderate. Family members frequently short-tempered with one another; and/or children in the home getting in physical fights with one another.
- Severe. Family members frequently short-tempered with one another and adults in the home throwing things at one another, and/or knocking over furniture, and/or hitting and/or harming one another.

What has been your experience of stress related to the COVID-19 pandemic?

- None.
- Mild. Occasional worries and/or minor stress-related symptoms (e.g., feel a little anxious, sad, and/or angry; mild/rare trouble sleeping).
- Moderate. Frequent worries and/or moderate stress-related symptoms (e.g., feel moderately anxious, sad, and/or angry; moderate/occasional trouble sleeping).
- Severe. Persistent worries and/or severe stress-related symptoms (e.g., feel extremely anxious, sad, and/or angry; severe/frequent trouble sleeping).

In general, what has been your **overall** stress level related to COVID-19?

No stress	1	2	3	4	5	6	7	8	9	10 (extreme stress)

If you are concerned that you may be a victim of domestic violence, or just to obtain more information, the following links contain lists of useful resources:

Across Canada:

- Ending Violence - Association of Canada. Links for each province or territory
- Status of Women Canada - Crisis lines for each province or territory
- Canadian Resource Centre for Victims of Crime

Worldwide:

- In the United States of America: National Domestic Violence Hotline
- International: List of resources in various countries as maintained by the National Center on Domestic and Sexual Violence

Stress related to COVID-19 pandemic

How has COVID-19 changed your **sleep**?

- ◯ Worsened it significantly
- ◯ Worsened it moderately
- ◯ No change
- ◯ Improved it moderately
- ◯ Improved it significantly

Which of the following statements apply to what you have been doing because of COVID-19? (Check all that apply)

- ☐ Reduced in-person contact with family inside the home (in other words, you have decided to reduce some kinds of contact with one or more members of your household)
- ☐ Reduced in-person contact with family members who live outside the home
- ☐ Reduced in-person contact with friends
- ☐ Reduced in-person contact with colleagues at work
- ☐ Stopped going to in-person events in the community
- ☐ Stopped going to in-person religious services
- ☐ Avoided leaving the house for non-essential reasons
- ☐ Used social distancing (6 feet/2 meters from others) when out in public
- ☐ Wore a mask in public
- ☐ Avoided crowds and large gatherings
- ☐ Washed your hands more regularly
- ☐ Avoided touching your face
- ☐ Cancelled travel
- ☐ Worked from home

In the past week, how many **hours** have you been spending keeping up with the news about COVID-19 by watching TV reports, reading newspaper articles, listening to the news on the radio, or getting information from the internet?

[ ]

We would like to know how you've been feeling lately.

Overall, how has the COVID-19 pandemic impacted you and your family?

- ◯ Extremely positive
- ◯ Moderately positive
- ◯ Somewhat positive
- ◯ No impact
- ◯ Somewhat negative
- ◯ Moderately negative
- ◯ Extremely negative

Emotions during the COVID-19 pandemic

We would now like to ask you about your emotions. When answering the following questions, we ask that you think about how you have felt overall in the last 7 days, not just how you felt today.

Here is an example:
I have felt happy:
Yes, all the time
Yes, most of the time - This would mean: ‘I have felt happy most of the time during the past week’
No, not very often
No, not at all

I have been able to laugh and see the funny side of things.

As much as I always could	Not quite so much now	Definitely not so much now	Not at all
◯	◯	◯	◯

I have looked forward with enjoyment to things.

As much as I ever did	Rather less than I used to	Definitely less than I used to	Hardly at all
◯	◯	◯	◯

I have blamed myself unnecessarily when things went wrong.

Yes, most of the time	Yes, some of the time	Not very often	No, never
◯	◯	◯	◯

I have been anxious or worried for no good reason.

No, not at all	Hardly ever	Yes, sometimes	Yes, very often
◯	◯	◯	◯

I have felt scared of panicky for no good reason.

Yes, quite a lot	Yes, sometimes	No, not so much	No not at all
◯	◯	◯	◯

Things have been getting on top of me.

Yes, most of the time I haven't been able to cope at all	Yes, sometimes I haven't been coping as well as usual	No, most of time I have coped quite well	No, I have been coping as well as ever
◯	◯	◯	◯

I have been so unhappy that I have had difficulty sleeping.

Yes, most of the time	Yes, sometimes	Not very often	No not at all
○	○	○	○

I have felt sad or miserable.

Yes, most of the time	Yes, sometimes	Not very often	No not at all
○	○	○	○

I have been so unhappy that I have been crying.

Yes, most of the time	Yes, quite often	Only occasionally	No, never
○	○	○	○

The thought of harming myself has occurred to me.

Yes, quite often	Sometimes	Hardly ever	Never
○	○	○	○

If you are concerned for your well-being, or simply to obtain more information, the following links offer lists of resources available to you:

Across Canada:

- Crisis Services Canada. Specific local support
- Canadian Crisis Centers Canada-wide and by province/territory centers
- Suicide Action Montreal - now for all of the Province of Quebec

Worldwide:

- International Association for Suicide Prevention - Resources: Crisis Centers
- International Crisis Hotlines - a list maintained by the LifeLine Canada Foundation
- United States of America Crisis Centers - list maintained by The LifeLine Canada Foundation

Anxiety during the COVID-19 pandemic

When answering the following questions, we ask that you think about how you have felt overall in the last 2 weeks, not just how you felt today.

During the last **2 weeks**, how often have you been bothered by the following problems:

	Not at all sure	Several days	Over half the days	Nearly every day
...feeling nervous, anxious or on edge.	◯	◯	◯	◯
...not being able to stop of control worrying.	◯	◯	◯	◯
...worrying too much about different things.	◯	◯	◯	◯
...trouble relaxing.	◯	◯	◯	◯
...being so restless that it's hard to sit still.	◯	◯	◯	◯
...becoming easily annoyed or irritable.	◯	◯	◯	◯
...feeling afraid as if something awful might happen.	◯	◯	◯	◯

If you have checked off any problems, how difficult have these made it for you to do your work, take care of things at home, or get along with other people?

- ◯ Not difficult at all.
- ◯ Somewhat difficult.
- ◯ Very difficult.
- ◯ Extremely difficult.

Lifestyle habits

What type of physical activity do you engage in (more than 2 times a week) **during** your pregnancy? (Check all that applies)

- ☐ None at all
- ☐ Cycling
- ☐ Gym/workout
- ☐ Walking
- ☐ Gardening
- ☐ Yoga
- ☐ Swimming
- ☐ Dancing
- ☐ Other

How has your physical activity changed due to the COVID-19 pandemic?

- ○ No change
- ○ I started exercising
- ○ I stopped exercising
- ○ I increased my physical activity
- ○ I decreased my physical activity

Are you currently drinking caffeinated beverages (Pepsi®, Coca Cola®, coffee, tea, energy drinks)?

- ○ Yes
- ○ Prefer not to answer
- ○ No

How has your caffeine consumption changed due to the COVID-19 pandemic?

- ○ No change
- ○ I started drinking caffeinated beverages
- ○ I stopped drinking caffeinated beverages
- ○ I increased my intake of caffeinated beverages
- ○ I decreased my intake of caffeinated beverages

Smoking habits

Are you **currently** smoking?

- ◯ Yes
- ◯ No
- ◯ Prefer not to answer

How has your **smoking** status changed since the COVID-19 pandemic?

- ◯ No change
- ◯ I **started** smoking
- ◯ I **stopped** smoking
- ◯ I smoke **less** than I did before
- ◯ I smoke **more** than I did before

Alcohol consumption

Are you **currently** drinking alcoholic beverages (wine, beer, spirits, etc)?

- ◯ Yes
- ◯ No
- ◯ Prefer not to answer

How have your **drinking habits** changed since the COVID-19 pandemic?

- ◯ No change
- ◯ I **started** drinking alcoholic beverages
- ◯ I **stopped** drinking alcoholic beverages
- ◯ I drink **less** alcoholic beverages than I did before
- ◯ I drink **more** alcoholic beverages than I did before

Cannabis consumption

Are you **currently smoking** cannabis products?

- Yes
- No
- Prefer not to answer

How has your **cannabis smoking** changed since the COVID-19 pandemic?

- No change
- I **started** smoking cannabis products
- I **stopped** smoking cannabis products
- I smoke cannabis products **less** than I did before
- I smoke cannabis products **more** than I did before

Are you **currently** using cannabis products in alternative forms (e.g. oils, edibles)?

- Yes
- No
- Prefer not to answer

How has your use of **alternative cannabis products** changed since the COVID-19 pandemic?

- No change
- I **started** using alternative cannabis products
- I **stopped** using alternative cannabis products
- I use alternative cannabis products **less** than I did before
- I use alternative cannabis products **more** than I did before

Illicit drug use

Are you **currently** using illicit drugs (cocaine, speed, heroin, etc)?

- Yes
- No
- Prefer not to answer

How has your **use of illicit drugs** changed since the COVID-19 pandemic?

- No change
- I **started** using illicit drugs
- I **stopped** using illicit drugs
- I use **less** illicit drugs than I did before
- I use **more** illicit drugs than I did before

Multivitamin use

Were you taking multivitamins (e.g. Centrum®) **before** becoming pregnant?

- ◯ Yes
- ◯ No

Are you **currently** taking prenatal multivitamins (e.g. Materna®, Centrum®)?

- ◯ Yes
- ◯ No

Medical conditions and medication use

Has a physician diagnosed you with any of the following conditions? (Check all that apply)

- [ ] Asthma
- [ ] Nausea
- [ ] High blood pressure
- [ ] Thyroid disease
- [ ] Diabetes
- [ ] High Cholesterol
- [ ] Chronic migraines
- [ ] Cancer
- [ ] Epilepsy
- [ ] Heart disease
- [ ] Other, please specify.
- [ ] Lung disease
- [ ] Anemia or other blood disease
- [ ] Ulcer or stomach disease
- [ ] Kidney disease
- [ ] Liver disease
- [ ] Pain
- [ ] Flu/Influenza
- [ ] Infection
- [ ] None

Are you using **prescribed treatments** for any of the following conditions? (Check all that apply)

- [ ] Asthma
- [ ] Nausea
- [ ] High blood pressure
- [ ] Thyroid disease
- [ ] Diabetes
- [ ] High Cholesterol
- [ ] Chronic migraines
- [ ] Cancer
- [ ] Epilepsy
- [ ] Heart disease
- [ ] Other, please specify.
- [ ] Lung disease
- [ ] Anemia or other blood disease
- [ ] Ulcer or stomach disease
- [ ] Kidney disease
- [ ] Liver disease
- [ ] Pain
- [ ] Flu/Influenza
- [ ] Infection
- [ ] None

Over-the-counter medications

Are you **currently** using over-the-counter medications (those that do **not** require a doctor’s prescription)?

- ☐ Tylenol®/Acetaminophen/Paracetamol
- ☐ Aspirin®/Baby Aspirin
- ☐ Pepto-Bismol®/TUMS®/Bismuth subsalicylate/Calcium carbonate
- ☐ Cough syrup/lozenges
- ☐ Gravol®/Dimenhydrinate/Dramamine
- ☐ Exlax®/Senekot/Laxatives
- ☐ Advil®/Motrin®/Nurofen/Ibuprofen
- ☐ Imodium®/Loperamide
- ☐ Robaxin®/Robaxacet®/Tylenol® Body Pain Night/Robax® Platinum/Methocarbamol
- ☐ Aleve®/Naproxen
- ☐ None
- ☐ Other

YOU ARE INVITED: To be **contacted for a follow-up questionnaire** 2 months following your delivery

Thank you for answering this questionnaire.

Would you be interested in filling up a second (similar but shorter) questionnaire at 2 months following your delivery? This follow-up questionnaire would inform us on the changes to your health as well as provide us information with the health of your baby, as well as your delivery or your breastfeeding habits, and will take approximately 15 minutes. We will contact you according to your preferred means of communication.

- ◯ No
- ◯ Yes

YOU'VE SAID YES: To be contacted for a follow-up questionnaire 2 months following your delivery

What is your name?

First name

Last name

If you wish to be contacted by phone:

Phone Number

If you wish to be contacted by **email**:

Please enter your email address.

Please confirm your email address.

YOU ARE INVITED: Confidential linkage of personal data

We would like to know more about your use of health services (eg. consultations in hospitals, clinics and doctors' offices) during and after pregnancy, and of your newborn baby. To do this, we ask your permission to link the data collected in this questionnaire with administrative health data from your province: provincial databases of medical services utilization, pharmaceutical services utilization and prescription filling at the pharmacy, hospitalizations, and emissions of birth and death certificates. This will allow us to receive information about the medicines and health services you have used after completing this reference questionnaire.

This linked information will be kept strictly confidential and will only be used for research purposes. A refusal will not change the quality or quantity of the health care or services you receive or to which you have the right.

If you agree, a designated person on the research team will forward your first name, last name, date of birth and provincial health card number to link your survey data to your health data.

All information transfers will be made by registered mail and secure computer files. The research team will then link the databases. In order to preserve your identity and the confidentiality of your personal information, all information allowing you to be identified will subsequently be erased from the database and you will be identified only by a code number.

* Do you give us permission to do this match?

- ◯ Yes
- ◯ No

If **no**, please share your reason(s):

[ ]

YOU'VE SAID YES: **Confidential linkage** of personal data

* Please provide the following information:

First Name	
Last Name	
Peronsal health insurance number as it appears on your provincial health insurance card for example (without spaces)	

* Please confirm your date of birth.

	Day	Month	Year
Birth date			

CONCEPTION Study

You are being redirected

Thank you so much for your interest!

Please click the following link to redirect you to the proper survey!

Survey for women who delivered between March 13th and today.

Thank you *very much* for your time!

You have now finished. Thank you for answering this questionnaire. We assure you that this data will remain confidential. If you have questions, do not hesitate to ask the study coordinator for help.

Yessica-Haydee Gomez, MSc.
Study coordinator
yessica-haydee.gomez@recherche-ste-justine.qc.ca
514-345-4931 ext. 4271
1-866-220-2654 (toll-free)

CONCEPTION Study

Thank you for your interest!

CONCEPTION Study: Short- and long-term impact of COVID-19 public health guidelines and hospital policies on maternal and child mental and physical health.

Thanks anyways for your interest!
To know how representative our participants are of all the people who have heard of our project, we invite you to answer the following 2 questions:

How old are you?

Reason for refusal to participate
