# Supplementary material for: Maternal Mental Health in Pregnancy and Its Impact on Children’s Cognitive Development at 18 Months, during the COVID-19 Pandemic (CONCEPTION Study)
Source: J Clin Med. 2024 Feb 13;13(4):1055. doi: 10.3390/jcm13041055 (PMC10889100; doi:10.3390/jcm13041055)
Supplement: Supplementary file 1 [file jcm-13-01055-s001.zip › Questionnaires/Q2.pdf]

CONCEPTION Study - Postpartum Questionnaire

WELCOME BACK TO THE CONCEPTION STUDY!

We want to **thank you** for agreeing to fill out this postpartum questionnaire. We are interested in information relating to your physical and mental health, and that of your baby.

We encourage you to fill out this questionnaire online, but, if you prefer, you can complete it by phone by calling our team by dialing:

- In Montreal (Quebec, Canada) and surroundings: 514 345-4931, extension 7780
- Elsewhere in the world: 1-866-220-2654 (toll free)

Please leave your name, your phone number, and the best time at which to reach you if we are unable to answer.

This questionnaire takes approximately 15 minutes to complete.

Let's begin.

General and Socio-demographic Information

* Please state your full name:

First Name [ ]

Last Name [ ]

* Please confirm your date of birth.

	Day	Month	Year
Birth date			

What was the outcome of your pregnancy?

- ◯ Livebirth
- ◯ Stillbirth
- ◯ Abortion
- ◯ Miscarriage
- ◯ Death following delivery

CONCEPTION Study - Postpartum Questionnaire

General and Socio-demographic Information

At how many weeks of pregnancy did you deliver?

Has a health professional told you that you have postpartum depression?

- No
- Yes
- Prefer not to answer

If yes, at how many weeks following your delivery?

Personal Experience with COVID-19

Since filling out the first questionnaire for this study, have you **been tested** for COVID-19?

- ◯ Yes
- ◯ No

If yes, **which type** of test did you receive? (check all that apply)

- ☐ Nasal/throat swab
- ☐ Blood test
- ☐ Based on symptoms and fever only

Since filling out the first questionnaire for this study, have you **tested positive** on a COVID-19 test?

- ◯ Yes
- ◯ No

If yes, **when** was this?

- ◯ During my pregnancy
- ◯ At delivery
- ◯ After delivery, but during my hospital stay
- ◯ After delivery, but once I was discharged from the hospital

Personal Experience with COVID-19

If you **believe** that you have had COVID-19, how severe was it?

- ◯ None. I had no symptoms
- ◯ Mild. Symptoms effectively managed at home
- ◯ Moderate. Symptoms severe and required brief hospitalization
- ◯ Severe. Symptoms severe and required ventilation (admission to intensive care and requiring intubation)

If you believe that you have had COVID-19, have you **recovered**?

- ◯ Yes, totally
- ◯ Partially
- ◯ Not at all

CONCEPTION Study - Postpartum Questionnaire

Pregnancy experience related to the COVID-19 pandemic

Did the support you receive from your primary prenatal care provider(s) change due to the COVID-19 pandemic?

- ◯ Significantly worsened
- ◯ Somewhat worsened
- ◯ No change
- ◯ Somewhat improved
- ◯ Significantly improved

Have you experienced a change in the type of prenatal classes/information as a result of the COVID-19 pandemic? (check all that apply)

- ☐ Not applicable: I had not been receiving any prenatal education before COVID-19
- ☐ No change in my access to prenatal education
- ☐ My classes were cancelled completely
- ☐ My classes changed to online/virtual classes
- ☐ My class was replaced by reading material only
- ☐ I went online to get prenatal education and information
- ☐ Other

Pregnancy

During pregnancy, did you experience any of the following? (check all that apply)

- [ ] Gestational diabetes
- [ ] Hypertension
- [ ] Preeclampsia
- [ ] Short cervix
- [ ] Small fetal size
- [ ] Bleeding or spotting
- [ ] Other
- [ ] None of these apply

During your delivery, did you experience any of the following? (check all that apply)

- [ ] Preeclampsia
- [ ] Use of oxytocin
- [ ] Use of forceps
- [ ] Use of vacuum extraction
- [ ] Hemorrhage (excessive blood loss)
- [ ] Delivery <32 weeks gestation
- [ ] Delivery <37 weeks gestation
- [ ] Low birth weight
- [ ] Other
- [ ] None of these apply

CONCEPTION Study - Postpartum Questionnaire

Labour/Birth

Did any of the following happen during or following your labor and delivery because of the COVID-19 pandemic? (check all that apply)

- [ ] Reduced access to preferred medications before or after delivery (i.e. nitrous oxide, epidural)
- [ ] I was not able to have a water birth
- [ ] My elective induction or C-section was not permitted as planned
- [ ] My health care provider (e.g., doctor, doula, midwife) was not available by my baby's birth as planned
- [ ] Support people (e.g., partner, family member) were not permitted to attend baby's delivery
- [ ] I did not have the support I needed to have the most natural delivery I was planning for (e.g., bath, massage, walking, pressure points, ball, etc.)
- [ ] I was separated from my baby immediately after delivery
- [ ] I was not allowed to keep my baby in my room
- [ ] I was sent home early after labor (i.e. shorter stay in hospital than planned)
- [ ] I had birth complications due to contracting COVID-19
- [ ] I was exposed to COVID-19 during pregnancy
- [ ] I was exposed to COVID-19 during labor/delivery or shortly thereafter
- [ ] I was not provided adequate opportunity for immediate skin-to skin contact with my newborn
- [ ] I was not provided adequate opportunity to try to initiate breastfeeding
- [ ] Family and friends were not able to visit me and my baby (e.g., due to social distancing or travel restrictions)
- [ ] My child became infected with COVID-19 after birth
- [ ] I became infected with COVID-19 and was unable to care for my baby
- [ ] My baby received less optimal postnatal care due to the COVID-19 pandemic (e.g., fewer checkups after birth)
- [ ] Other
- [ ] None of these apply

Where did you give birth?

- ◯ Hospital
- ◯ Birth centre located in a hospital
- ◯ Birthing/maternity house (**not** in a hospital)
- ◯ Home
- ◯ On the way to the hospital or birth centre or birthing/maternity house
- ◯ Other

What was your mode of delivery?

- ◯ Vaginal
- ◯ Caesarean, planned
- ◯ Caesarean, urgent

How long did you remain at the hospital after your delivery?

- ◯ 2 days or less
- ◯ More than 2 days

If more than 2 days, what was the reason?

Delivery

During your delivery, did your baby experience any of the following? (check all that apply)

- ☐ Baby sent to neonatal intensive care unit (NICU) or pediatric intensive care unit (PICU)
- ☐ Baby’s heartbeat was too low
- ☐ The baby had jaundice
- ☐ The baby needed extra care after birth (e.g., resuscitation)
- ☐ The baby had a malformation or physical anomaly
- ☐ None of the above

Did your newborn baby leave the hospital at the same time as you?

- ◯ Yes
- ◯ No

If no, what was the reason?

Experience in the neonatal intensive care unit (NICU) or pediatric intensive care unit (PICU)

If a stay in neonatal intensive care unit (NICU) or pediatric intensive care unit (PICU) was necessary, were you allowed to visit your newborn baby in NICU or PICU?

- ◯ Not applicable
- ◯ No
- ◯ Yes

Your Newborn(s)

Did you have a single (one newborn baby), twin (two newborn babies) or multiple pregnancy (three or more newborn babies)?

- ◯ Single
- ◯ Twin
- ◯ Multiple

If multiple, how many?

[ ]

CONCEPTION Study - Postpartum Questionnaire

Your Newborn - Singleton Pregnancy

What is the sex of your newborn?

- ◯ Male
- ◯ Female
- ◯ Other (please specify)

Weight at birth

Enter weight

Was a diagnosis of any malformations made in your newborn baby?

- ◯ No
- ◯ Yes

If yes, please describe

Was your newborn baby diagnosed with COVID-19?

- ◯ No
- ◯ Yes, at delivery
- ◯ Yes, after delivery

Which of the following best describes the overall health of your baby?

- ◯ Healthy
- ◯ Minor health concerns
- ◯ Major health concerns
- ◯ Prefer not to answer

CONCEPTION Study - Postpartum Questionnaire

Your Newborn - Baby 1 - Twin or Multiple Pregnancy

Please enter the information concerning Baby 1 (follow the order of birth).

What is the sex of your newborn?

- ◯ Male
- ◯ Female
- ◯ Other (please specify)

Weight at birth

Enter weight

Was a diagnosis of any malformations made in your newborn baby?

- ◯ No
- ◯ Yes

If yes, please describe

Was your newborn baby diagnosed with COVID-19?

- ◯ No
- ◯ Yes, at delivery
- ◯ Yes, after delivery

Which of the following best describes the overall health of your baby?

- ◯ Healthy
- ◯ Minor health concerns
- ◯ Major health concerns
- ◯ Prefer not to answer

CONCEPTION Study - Postpartum Questionnaire

Your Newborn - Baby 2 - Twin or Multiple Pregnancy

Please enter the information concerning Baby 2 (follow the other of birth).

What is the sex of your newborn?

- Male
- Female
- Other (please specify)

Weight at birth

Enter weight

Was a diagnosis of any malformations made in your newborn baby?

- No
- Yes

If yes, please describe

Was your newborn baby diagnosed with COVID-19?

- No
- Yes, at delivery
- Yes, after delivery

Which of the following best describes the overall health of your baby?

- ◯ Healthy
- ◯ Minor health concerns
- ◯ Major health concerns
- ◯ Prefer not to answer

Did you have more than 2 babies?

- ◯ Yes
- ◯ No

CONCEPTION Study - Postpartum Questionnaire

Your Newborn - Baby 3 - Multiple Pregnancy

Please enter the information concerning Baby 3 (follow the other of birth).

What is the sex of your newborn?

- ◯ Male
- ◯ Female
- ◯ Other (please specify)

Weight at birth

Enter weight

Was a diagnosis of any malformations made in your newborn baby?

- ◯ No
- ◯ Yes

If yes, please describe

Was your newborn baby diagnosed with COVID-19?

- ◯ No
- ◯ Yes, at delivery
- ◯ Yes, after delivery

Which of the following best describes the overall health of your baby?

- ◯ Healthy
- ◯ Minor health concerns
- ◯ Major health concerns
- ◯ Prefer not to answer

Did you have more than 3 babies?

- ◯ Yes
- ◯ No

Your Newborn - Baby 4 - Multiple Pregnancy

Please enter the information concerning Baby 4 (follow the other of birth).

What is the sex of your newborn?

- ◯ Male
- ◯ Female
- ◯ Other (please specify)

Weight at birth

Enter weight

Was a diagnosis of any malformations made in your newborn baby?

- ◯ No
- ◯ Yes

If yes, please describe

Was your newborn baby diagnosed with COVID-19?

- ◯ No
- ◯ Yes, at delivery
- ◯ Yes, after delivery

Which of the following best describes the overall health of your baby?

- ◯ Healthy
- ◯ Minor health concerns
- ◯ Major health concerns
- ◯ Prefer not to answer

CONCEPTION Study - Postpartum Questionnaire

Breastfeeding

Are you currently breastfeeding your baby?

- ◯ Yes
- ◯ No
- ◯ Prefer not to answer

How do you feed your baby?

- ◯ Breastfeeding or Mother's expressed breast milk only
- ◯ Formula only
- ◯ Combination of breastfeeding/mother's expressed milk and formula

CONCEPTION Study - Postpartum Questionnaire

Maternal Care

Did any of your experiences after your delivery change because of the COVID-19 pandemic? (check all that apply)

- [ ] I did not have access to lactation support or other types of support following discharge from the hospital
- [ ] My postpartum visit was a virtual visit
- [ ] My postpartum visit was cancelled
- [ ] My postpartum visit was postponed
- [ ] I was unable to get the type of contraception that I wanted
- [ ] I was unable to discuss “baby blues” or issues related to my mood
- [ ] My baby’s well visits were made virtual
- [ ] My baby’s well visits were canceled
- [ ] My baby's well visits were postponed
- [ ] My baby’s immunizations were postponed
- [ ] No change

Perinatal Experience

In general, what has been your level of experienced distress with regards to changes in your birth and postnatal care due to COVID-19?

No distress						Highly distressed
○	○	○	○	○	○	○

CONCEPTION Study - Postpartum Questionnaire

Satisfaction with Life

We are now going to talk about the quality of your life in relation to both your pregnancy and personal health.

In general, would you say your health is:

- ◯ Excellent
- ◯ Very good
- ◯ Good
- ◯ Fair
- ◯ Poor

Below are five statements that you may agree or disagree with. Using the 1 - 7 scale below, indicate your agreement with each item. Please be open and honest in your response.

	Strongly disagree 1	Disagree 2	Slightly disagree 3	Neither agree nor disagree 4	Slightly agree 5	Agree 6	Strongly agree 7
In most ways my life is close to my ideal.	◯	◯	◯	◯	◯	◯	◯
The conditions of my life are excellent.	◯	◯	◯	◯	◯	◯	◯
I am satisfied with my life.	◯	◯	◯	◯	◯	◯	◯
So far I have gotten the important things I want in life.	◯	◯	◯	◯	◯	◯	◯
If I could live my life over, I would change almost nothing.	◯	◯	◯	◯	◯	◯	◯

Stress Related to the COVID-19 Pandemic

Stress and discord in the family or with other people you live with:

- ○ No change
- ○ Mild. Family members occasionally short-tempered with one another; no physical violence
- ○ Moderate. Family members frequently short-tempered with one another; and/or children in the home getting in physical fights with one another
- ○ Severe. Family members frequently short-tempered with one another and adults in the home throwing things at one another, and/or knocking over furniture, and/or hitting and/or harming one another

What has been your experience of stress related to the COVID-19 pandemic?

- ○ None
- ○ Mild. Occasional worries and/or minor stress-related symptoms (e.g., feel a little anxious, sad, and/or angry; mild/rare trouble sleeping)
- ○ Moderate. Frequent worries and/or moderate stress-related symptoms (e.g., feel moderately anxious, sad, and/or angry; moderate/occasional trouble sleeping)
- ○ Severe. Persistent worries and/or severe stress-related symptoms (e.g., feel extremely anxious, sad, and/or angry; severe/frequent trouble sleeping)

In general, what has been your **overall** stress level related to COVID-19?

No stress	1	2	3	4	5	6	7	8	9	10
○	○	○	○	○	○	○	○	○	○	○

If you are concerned that you may be a victim of domestic violence, or just to obtain more information, the following links contain lists of useful resources:

Across Canada:

- Ending Violence - Association of Canada. Links for each province or territory
- Status of Women Canada - Crisis lines for each province or territory
- Canadian Resource Centre for Victims of Crime

Worldwide:

- In the United States of America: National Domestic Violence Hotline
- International: List of resources in various countries as maintained by the National Center on Domestic and Sexual Violence

Stress Related to the COVID-19 Pandemic

How has COVID-19 changed your **sleep**?

- Worsened it significantly
- Worsened it moderately
- No change
- Improved it moderately
- Improved it significantly

Which of the following statements apply to what you have been doing because of COVID-19? (check all that apply)

- [ ] Reduced in-person contact with family inside the home (in other words, you have decided to reduce some kinds of contact with one or more members of your household)
- [ ] Reduced in-person contact with family members who live outside the home
- [ ] Reduced in-person contact with friends
- [ ] Reduced in-person contact with colleagues at work
- [ ] Stopped going to in-person events in the community
- [ ] Stopped going to in-person religious services
- [ ] Avoided leaving the house for non-essential reasons
- [ ] Used social distancing (6 feet/2 meters from others) when out in public
- [ ] Wore a mask in public
- [ ] Avoided crowds and large gatherings
- [ ] Washed your hands more regularly
- [ ] Avoided touching your face
- [ ] Cancelled family or personal travel
- [ ] Worked from home
- [ ] Stopped going to restaurants or stores
- [ ] None of the above
- [ ] Other

Other (please specify)

In the past week, how many hours have you been spending keeping up with the news about COVID-19 by watching TV reports, reading newspaper articles, listening to the news on the radio, or getting information from the internet?

We would like to know how you've been feeling lately.

Overall, what has the COVID-10 pandemic impacted your family and you?

- ◯ No impact
- ◯ Extremely negative
- ◯ Moderately negative
- ◯ Somewhat negative
- ◯ Somewhat positive
- ◯ Moderately positive
- ◯ Extremely positive

CONCEPTION Study - Postpartum Questionnaire

Emotions during the COVID-19 Pandemic

AS YOU HAVE RECENTLY HAD A BABY, WE WOULD LIKE TO KNOW HOW YOU ARE FEELING NOW. Please choose the answer which comes closest to how you have felt IN THE PAST SEVEN DAYS, not just how you feel today.
Here is an example:

I have felt happy:

- Yes, all the time
- **Yes, most of the time** **->** This would mean: 'I have felt happy most of the time during the past week'
- No, not very often
- No, not at all

I have been able to laugh and see the funny side of things.

As much as I always could	Not quite so much now	Definitely not so much now	Not at all
○	○	○	○

I have looked forward with enjoyment to things.

As much as I ever did	Rather less than I used to	Definitely less than I used to	Hardly at all
○	○	○	○

I have blamed myself unnecessarily when things went wrong.

Yes, most of the time	Yes, some of the time	Not very often	No, never
○	○	○	○

I have been anxious or worried for no good reason.

No, not at all	Hardly ever	Yes, sometimes	Yes, very often
○	○	○	○

I have felt scared or panicky for no good reason.

Yes, quite a lot	Yes, sometimes	No, not so much	No not at all
○	○	○	○

Things have been getting on top of me.

Yes, most of the time I haven't been able to cope at all	Yes, sometimes I haven't been coping as well as usual	No, most of time I have coped quite well	No, I have been coping as well as ever
○	○	○	○

I have been so unhappy that I have had difficulty sleeping.

Yes, most of the time	Yes, sometimes	Not very often	No not at all
○	○	○	○

I have felt sad or miserable.

Yes, most of the time	Yes, sometimes	Not very often	No not at all
○	○	○	○

I have been so unhappy that I have been crying.

Yes, most of the time	Yes, quite often	Only occasionally	No, never
○	○	○	○

The thought of harming myself has occurred to me.

Yes, quite often	Sometimes	Hardly ever	Never
○	○	○	○

If you are concerned for your well-being, or simply to obtain more information, the following links offer lists of resources available to you:

Across Canada:

- Crisis Services Canada. Specific local support
- Canadian Crisis Centers Canada-wide and by province/territory centers
- Suicide Action Montreal - now for all of the Province of Quebec

Worldwide:

- International Association for Suicide Prevention - Resources: Crisis Centers
- International Crisis Hotlines - a list maintained by the LifeLine Canada Foundation
- United States of America Crisis Centers - list maintained by The LifeLine Canada Foundation

CONCEPTION Study - Postpartum Questionnaire

Anxiety during the COVID-19 Pandemic

When answering the following questions, we ask that you think about how you have felt overall in the last **2 weeks**, not just how you felt today.

During the last **2 weeks**, how often have you been bothered by the following problems?

	Not at all sure	Several days	Over half the days	Nearly every day
Feeling nervous, anxious or on edge.				
Not being able to stop of control worrying.				
Worrying too much about different things.				
Trouble relaxing.				
Being so restless that it's hard to sit still.				
Becoming easily annoyed or irritable.				
Feeling afraid as if something awful might happen.				

If you have checked off any problems, how difficult have these made it for you to do your work, take care of things at home, or get along with other people?

- ◯ Not difficult at all
- ◯ Somewhat difficult
- ◯ Very difficult
- ◯ Extremely difficult

CONCEPTION Study - Postpartum Questionnaire

We thank you for your interest

We are sorry for your loss.
Your follow-up questionnaire is now complete.

CONCEPTION Study - Postpartum Questionnaire

Thank you so much for your time!

You have now finished. Thank you for answering this questionnaire. We assure you that this data will remain confidential. If you have questions, do not hesitate to ask the study coordinator for help.

Yessica-Haydee Gomez, M.Sc.
Study coordinator
yessica-haydee.gomez@recherche-ste-justine.qc.ca
514-345-4931 ext. 4271
1-866-220-2654 (toll-free)
