# Supplementary material for: Maternal Mental Health in Pregnancy and Its Impact on Children’s Cognitive Development at 18 Months, during the COVID-19 Pandemic (CONCEPTION Study)
Source: J Clin Med. 2024 Feb 13;13(4):1055. doi: 10.3390/jcm13041055 (PMC10889100; doi:10.3390/jcm13041055)
Supplement: Supplementary file 1 [file jcm-13-01055-s001.zip › Questionnaires/Q3.pdf]

CONCEPTION Study - Postpartum Questionnaire 18 months

CONCEPTION Study: 18 months post-partum questionnaire

We are thankful that you are willing to participate in the CONCEPTION study once again.

I understand that the goal of Dr. Anick Bérard's research project is to collect and analyze responses to a short questionnaire on the impact of public and hospital health recommendations related to the COVID-19 pandemic on my mental and physical health. Through this questionnaire, the team is seeking to obtain longitudinal data in order to assess the impact of the pandemic on both the mother and the child as we enter another pandemic year.

This project has been approved by the Research Ethics Committee of the Research Center of the Center hospitalier universitaire (CHU) Sainte-Justine (Institutional ethical review approval number: **#2021-2973**).

* I agree to participate

- ◯ Yes
- ◯ No

CONCEPTION Study - Postpartum Questionnaire 18 months

Welcome back to the CONCEPTION Study!

This questionnaire should take approximately 20 minutes to complete, let's begin!

We will first ask you to confirm your identity with the following questions.

* Please state your full name.

First Name

Last Name

* Please confirm your date of birth.

	Day	Month	Year
Birth date			

Personal experience with depression and anxiety

Has a doctor diagnosed you with depression?

- ◯ No
- ◯ Yes, please enter the year of diagnosis

[ ]

Has a doctor diagnosed you with anxiety?

- ◯ No
- ◯ Yes, please enter the year of diagnosis

[ ]

Have you ever taken one of these medications? Select all that applies.

- ☐ Amitriptyline (Elavil)
- ☐ Bupropion (Wellbutrin)
- ☐ Citalopram (Celexa)
- ☐ Fluoxetine (Prozac)
- ☐ Sertraline (Zoloft)
- ☐ Venlafaxine (Effexor)
- ☐ Mirtazapine (Remeron)
- ☐ Escitalopram(Lexapro)
- ☐ Paroxetine(Paxil)
- ☐ Milnacipran (Savella)
- ☐ Duloxetine (Cymbalta)
- ☐ Fluvoxamine (Luvox)
- ☐ Reboxetine (Vestra)
- ☐ Diazepam (Valium)
- ☐ Alprazolam (Xanax)
- ☐ Lorazepam (Ativan)
- ☐ Clonazepam (Rivotril)
- ☐ Bromazepam (Lectopram, Lexomil)
- ☐ Oxazepam (Serax)
- ☐ Hydroxyzine dichlorhydrate (Atarax)
- ☐ Chlordiazepoxide (Librium)
- ☐ Etifoxine Chlorhydrate (Stresam)
- ☐ Clotiazepam (Veratran)
- ☐ Prazepam (Lysanxia)
- ☐ Clorazepate (Tranxene)
- ☐ Carbamazepine (Tegretol)
- ☐ Lamotrigine (Lamictal)
- ☐ Valproate (Depakene, Depival)
- ☐ Divalproex (Epival)
- ☐ Olanzapine (Zyprexa, Zydis)
- ☐ Quetiapine (Seroquel)
- ☐ Risperidone (Risperdal)
- ☐ Aripiprazole (Abilify)

CONCEPTION Study - Postpartum Questionnaire 18 months

Personal Experience with COVID-19

In the following section, we will be asking you about your COVID-19 history throughout the pandemic, starting from March 13th 2020 to today.

How many times have you been tested for COVID-19 since march 13th 2020, **using a PCR test**:

- ◯ 0-5
- ◯ 5-10
- ◯ 10+

How many times have you been tested for COVID-19 since march 13th 2020, **using an at home rapid test**:

- ◯ 0-5
- ◯ 5-10
- ◯ 10+

Did you receive a diagnosis of COVID-19 since filling up the last questionnaire? Select all that applies (could be more than once).

- ☐ No
- ☐ Yes, during my pregnancy
- ☐ Yes, after my delivery

Personal Experience with COVID-19

Enter the date of your COVID-19 diagnosis

Diagnosis 1 (if aplicable)

Date

DD/MM/YYYY

Diagnosis 2 (if applicable)

Date

DD/MM/YYYY

Diagnosis 3 (if applicable)

Date

DD/MM/YYYY

Did you receive treatments for COVID-19 ?

- No
- Yes

CONCEPTION Study - Postpartum Questionnaire 18 months

Personal Experience with COVID-19

Which treatments did you receive for COVID-19? Select all that apply

- [ ] Hydroxycholoroquine
- [ ] Chloroquine
- [ ] Azitromicyn
- [ ] Colchicine
- [ ] Remdesivir
- [ ] Lopinavir
- [ ] Ritonavir
- [ ] Favipiravir
- [ ] Oseltamivir
- [ ] Ivermectin
- [ ] Molnupiravir
- [ ] Baricitinib
- [ ] Tofacitinib
- [ ] Tocilizumab
- [ ] Sarilumab
- [ ] Hydrocortisone
- [ ] Heparin
- [ ] Enoxaparain
- [ ] Dalteparin
- [ ] Tinzaprin
- [ ] Interferon Beta-1a
- [ ] Interferon Beta-1b
- [ ] Interferon Alfa-2b
- [ ] Monoclonal antibodies – Sotorivimab, Bamlanivimab, Etesevimab, Carsirivimab, Imdevimab
- [ ] Convalescent plasma
- [ ] Angiotensin receptor blockers : Irbesartan, Valsartan, Losartan and Vandesartan
- [ ] ACE inhibitors : Benazepril, Captopril, Enalapril, Fosinopril, Lisinopril, Moexipril, Perindopril, Quinapril, Ramipril, Trandolapril
- [ ] Dexamthesaone
- [ ] Methylprednisolone
- [ ] Prednisolone
- [ ] Predinisone
- [ ] Zinc
- [ ] Vitamin D
- [ ] Vitamin C
- [ ] Other (please specify)

____

- [ ] None of the above

CONCEPTION Study - Postpartum Questionnaire 18 months

Personal Experience with COVID-19

Did you experience symptoms associated with your COVID-19 infection that lasted more than 2 weeks after the first symptom onset? Select all that applies and tell us how long the symptoms described lasted.

	Less than a month	2-3 months	4-6 months	7-12 months	More than a year	N/A
Difficulty breathing						
Shortness of breath						
Fatigue						
Loss of smell (partial or total)						
Loss of taste (partial or total)						
Distortion of smell (things don’t smell the same as you know them)						
Distortion of taste (things don’t taste the same as you know them						
Difficutly staying in a standing position						
Difficulty doing physical exercise						
Cough						
Joint pain						
Muscular pain						

Persistant fever	◯	◯	◯	◯	◯	◯
Fast or pounding heartbeat	◯	◯	◯	◯	◯	◯
Depression/anxiety	◯	◯	◯	◯	◯	◯
Loss of balance	◯	◯	◯	◯	◯	◯
Memory issues	◯	◯	◯	◯	◯	◯
Concentration issues	◯	◯	◯	◯	◯	◯
Sleep issues	◯	◯	◯	◯	◯	◯
Worsening symptoms after physical exercise	◯	◯	◯	◯	◯	◯

Other (please specify)

CONCEPTION Study - Postpartum Questionnaire 18 months

Personal experience with COVID-19 vaccines

In this section, we will inquire about COVID-19 vaccination.

Did you receive a COVID-19 vaccine?

- ◯ Yes, after I delivered
- ◯ Yes, during my pregnancy
- ◯ No

Personal experience with COVID-19 vaccines

Which vaccine did you receive?

- ◯ Pfizer/BioNTech
- ◯ Moderna
- ◯ Oxford University/AstraZeneca
- ◯ Johnson & Johnson
- ◯ I don't know
- ◯ Other (please specify)

How many doses did you receive so far?

- ◯ 1
- ◯ 2
- ◯ 3

Personal experience with COVID-19 vaccines

What are the reasons

- [ ] Public health advised against it
- [ ] Vaccines were not available to me
- [ ] Family doctor advised against it
- [ ] Obstetrician advised against it
- [ ] Nurse practitioner advised against it
- [ ] Midwife advised against it
- [ ] Lack of efficacy data in pregnancy or while breastfeeding
- [ ] Lack of safety data in pregnancy or while breastfeeding
- [ ] Lack of information on vaccines in pregnancy and post-partum
- [ ] The vaccine was created too quickly
- [ ] The vaccine was approved too quickly
- [ ] I've had COVID-19
- [ ] COVID-19 is not that serious
- [ ] I had lower exposure to COVID-19
- [ ] Other (please specify)

Stress Related to the COVID-19 Pandemic

In general, what has been your **overall** stress level related to COVID-19?

No stress	1	2	3	4	5	6	7	8	9	10
○	○	○	○	○	○	○	○	○	○	○

CONCEPTION Study - Postpartum Questionnaire 18 months

Emotions during the COVID-19 Pandemic

Please choose the answer which comes closest to how you have felt IN THE PAST SEVEN DAYS, not just how you feel today.
Here is an example:

I have felt happy:

- Yes, all the time
- **Yes, most of the time ->** This would mean: 'I have felt happy most of the time during the past week'
- No, not very often
- No, not at all

I have been able to laugh and see the funny side of things.

As much as I always could	Not quite so much now	Definitely not so much now	Not at all
◯	◯	◯	◯

I have looked forward with enjoyment to things.

As much as I ever did	Rather less than I used to	Definitely less than I used to	Hardly at all
◯	◯	◯	◯

I have blamed myself unnecessarily when things went wrong.

Yes, most of the time	Yes, some of the time	Not very often	No, never
◯	◯	◯	◯

I have been anxious or worried for no good reason.

No, not at all	Hardly ever	Yes, sometimes	Yes, very often
◯	◯	◯	◯

I have felt scared or panicky for no good reason.

Yes, quite a lot	Yes, sometimes	No, not so much	No not at all
◯	◯	◯	◯

Things have been getting on top of me.

Yes, most of the time I haven't been able to cope at all	Yes, sometimes I haven't been coping as well as usual	No, most of time I have coped quite well	No, I have been coping as well as ever
○	○	○	○

I have been so unhappy that I have had difficulty sleeping.

Yes, most of the time	Yes, sometimes	Not very often	No not at all
○	○	○	○

I have felt sad or miserable.

Yes, most of the time	Yes, sometimes	Not very often	No not at all
○	○	○	○

I have been so unhappy that I have been crying.

Yes, most of the time	Yes, quite often	Only occasionally	No, never
○	○	○	○

The thought of harming myself has occurred to me.

Yes, quite often	Sometimes	Hardly ever	Never
○	○	○	○

If you are concerned for your well-being, or simply to obtain more information, the following links offer lists of resources available to you:

Across Canada:

- Crisis Services Canada. Specific local support
- Canadian Crisis Centers Canada-wide and by province/territory centers
- Suicide Action Montreal - now for all of the Province of Quebec

Worldwide:

- International Association for Suicide Prevention - Resources: Crisis Centers
- International Crisis Hotlines - a list maintained by the LifeLine Canada Foundation
- United States of America Crisis Centers - list maintained by The LifeLine Canada Foundation

CONCEPTION Study - Postpartum Questionnaire 18 months

Anxiety during the COVID-19 Pandemic

When answering the following questions, we ask that you think about how you have felt overall in the last **2 weeks**, not just how you felt today.

During the last **2 weeks**, how often have you been bothered by the following problems?

	Not at all sure	Several days	Over half the days	Nearly every day
Feeling nervous, anxious or on edge.	○	○	○	○
Not being able to stop of control worrying.	○	○	○	○
Worrying too much about different things.	○	○	○	○
Trouble relaxing.	○	○	○	○
Being so restless that it's hard to sit still.	○	○	○	○
Becoming easily annoyed or irritable.	○	○	○	○
Feeling afraid as if something awful might happen.	○	○	○	○

If you have checked off any problems, how difficult have these made it for you to do your work, take care of things at home, or get along with other people?

- ◯ Not difficult at all
- ◯ Somewhat difficult
- ◯ Very difficult
- ◯ Extremely difficult

Child's well-being

In the next sections, we will be asking you about your child's health. If you have participated to our study more than once, we ask you to complete the following sections pertaining to the child you would have had about 18 months ago.

What is your child's current head circumference (in cm)?

Please measure the circumference of the head with a measuring tape, placing the tape above the eyebrows and behind the head, at the point of the skull, then return to the forehead.

Has a doctor or professional ever told you that your baby has one (or more) of the following conditions? Select all that applies.

- ☐ Blind or partially blind
- ☐ Nystagmus
- ☐ Strabismus
- ☐ Partial or complete deafness
- ☐ Abnormal muscle tone/muscle weakness
- ☐ Is gaining weight too slowly
- ☐ Is gaining weight too quickly
- ☐ Vitamins or minerals deficiencies
- ☐ Difficulties to be fed
- ☐ Cardiac anomaly confirmed by cardiac ultrasound
- ☐ Allergies
- ☐ Food intolerance
- ☐ Stridor
- ☐ Apnea
- ☐ Asthma
- ☐ Stomach infection or frequent diarrhea
- ☐ Kawasaki syndrome
- ☐ Syndrome or condition that affect brain development (e.g. Fragile X syndrome, Down Syndrome, copy number variation, Neurofibromatosis)
- ☐ Syndrome or condition that affect heart development (e.g. cardiopathy)
- ☐ Brain injury
- ☐ Cancer
- ☐ Other (please specify)

Has your child had a COVID-19 infection?

- ○ No
- ○ Yes

CONCEPTION Study - Postpartum Questionnaire 18 months

Child's well-being - COVID-19 experience

Has your child been hospitalized due to a COVID-19 infection?

- ◯ No
- ◯ Yes

Has your child had seizures (involuntary movements of all or part of the body's limbs) associated with COVID-19 infection?

- ◯ No
- ◯ Yes

Child's well-being - COVID-19 experience

Please answer the following questions thinking of the seizure episode in the context of a COVID-19 infection only.

What was the approximate date of first episode?

Date of first epidsode

Date

DD/MM/YYYY

Number of episodes?

Your child's body temperature during seizure episodes?

What was the approximate duration (in minutes) of the longest seizure episode?

Your child's body temperature during the **longest** seizure episode?

Has your child had more than one seizure during the same fever episode?

- No
- Yes

Did your child have seizures limited to one part of the body (e.g. one limb, half of the body)?

- ◯ No
- ◯ Yes

Child's health

Has your child ever been hospitalized?

- ◯ No
- ◯ Yes, for what reason (s)

[ ]

Two to five percent of children have a seizure by age 5. We want to know if your child has had a seizure (not associated with COVID-19).

Since birth, has your doctor confirmed that your child has had a seizure(s)?

- ◯ No
- ◯ Yes

Child's experience with seizures

Were the seizure episodes related to a fever episode (unlreated to a COVID-19 infection)?

- No
- Yes

What was the approximate date of **first episode**?

Date of first epidsode

Date

DD/MM/YYYY

Number of episodes?

What was the approximate date of the **last episode**?

Date of first epidsode

Date

DD/MM/YYYY

What is your child's body temperature during a typical seizure episode?

What was the approximate duration (in minutes) of the longest seizure episode?

Your child's body temperature during the **longest** seizure episode?

Has your child had more than one seizure during the same fever episode?

- ◯ No
- ◯ Yes

Did your child have seizures limited to one part of the body (e.g. one limb, half of the body)?

- ◯ No
- ◯ Yes

Has a doctor confirmed that your child has a seizure disorder or epilepsy?

- ◯ No
- ◯ Yes, seizure disorder
- ◯ Yes, epilepsy

CONCEPTION Study - Postpartum Questionnaire 18 months

Your child's development

In the following sections, we will be asking you questions about activities your child may or may not do. Your child may have done some of the activities described here and there may be some your child has not begun doing yet. For each item, please tell us if the activity is done regularly, sometimes or not yet.

Important to remember:

- Try each activity with your child before marking a response
- Make completing this questionnaire a game that is fun for you and your child
- Make sure your child is rested and fed
- We are so thankful for your participation!

At this age, many toddlers may not be cooperative when asked to do things. You may need to try the following activities with your child more than one time. If possible, try the activities when your child is cooperative. If your child can do the activity but refuses, mark "yes" for the item.

Communication

	Yes	Sometimes	Not yet
When your child wants something, does she tell you by pointing to it?	○	○	○
When you ask your child to, does he go into another room to find a familiar toy or object? (You might ask, "Where is your ball?" or say, "Bring me your coat," or "Go get your blanket.")	○	○	○
Does your child say eight or more words in addition to "Mama" and "Dada"?	○	○	○

Does your child imitate a two-word sentence? For example, when you say a two-word phrase, such as "Mama eat," "Daddy play," "Go home," or "What's this?" does your child say both words back to you? (Mark "yes" even if words are difficult to understand.)	◯	◯	◯
Without your showing them, does your child point to the correct picture when you say, "Show me the kitty," or ask, "Where is the dog?" (They need to identify only one picture correctly.)	◯	◯	◯
Does your child say two or three words that represent different ideas together, such as "See dog," "Mommy come home," or "Kitty gone"? (Don't count word combinations that express one idea, such as "bye-bye," "all gone," "all right," and "What's that?")	◯	◯	◯

Please give us an example of your child's word combinations:

[ ]

CONCEPTION Study - Postpartum Questionnaire 18 months

Your child's development

Please answer the following questions based on what describes your child best.

Gross Motor

	Yes	Sometimes	Not yet
Does your child bend over or squat to pick up an object from the floor and then stand up again without any support?	○	○	○
Does your child move around by walking, rather than by crawling on her hands and knees?	○	○	○
Does your child walk well and seldom fall?	○	○	○
Does your child climb on an object such as a chair to reach something they want (for example, to get a toy on a counter or to "help" you in the kitchen)?	○	○	○
Does your child walk downstairs if you hold onto one of their hands? They may also hold onto the	○	○	○

railing or wall. You can look for this at a store, on a playground, or at home.)			
When you show your child how to kick a large ball, do they try to kick the ball by moving their leg forward or by walking into it? (If your child already kicks a ball, mark "yes" for this item.)	◯	◯	◯

Fine Motor

	Yes	Sometimes	Not yet
Does your child throw a small ball with a forward arm motion? (If they simply drop the ball, mark "not yet" for this item.)	○	○	○
Does your child stack a small block or toy on top of another one?	○	○	○
Does your child make a mark on the paper with the tip of a crayon (or pencil or pen) when trying to draw?	○	○	○
Does your child stack three small blocks or toys on top of each other by themself?	○	○	○
Does your child turn the pages of a book by themself? (They may turn more than one page at a time.)	○	○	○
Does your child get a spoon into her mouth right side up so that the food usually doesn't spill?	○	○	○

CONCEPTION Study - Postpartum Questionnaire 18 months

Your child's development

Problem Solving

	Often or always	Sometimes	Rarely or never
Does your child drop several small toys, one after another, into a container like a bowl or box? (You may show them how to do it.)	○	○	○
After you have shown your child how, do they try to get a small toy that is slightly out of reach by using a spoon, stick, or similar tool?	○	○	○
After a crumb or Cheerio is dropped into a small, clear bottle, does your child turn the bottle over to dump it out? You may show them how.)	○	○	○
Without your showing them how, does your child scribble back and forth when you give them a crayon (or pencil or pen)?	○	○	○
After watching			

you draw a line from the top of the paper to the bottom with a crayon (or pencil or pen), does your child copy you by drawing a single line on the paper in any direction? (Mark "not yet" if your child scribbles back and forth.)	○	○	○
After a crumb or Cheerio is dropped into a small, clear bottle, does your child turn the bottle upside down to dump out the crumb or Cheerio? (Do not show them how.)	○	○	○

Personal-Social

	Yes	Sometimes	Not yet
While looking at themself in the mirror, does your child offer a toy to their own image?	○	○	○
Does your child play with a doll or stuffed animal by hugging it?	○	○	○
Does your child get your attention or try to show you something by pulling on your hand or clothes?	○	○	○
Does your child come to you when they need help, such as with winding up a toy or unscrewing a lid from a jar?	○	○	○
Does your child drink from a cup or glass, putting it down again with little spilling?	○	○	○
Does your child copy the activities you do, such as wipe up a spill, sweep, shave, or comb hair?	○	○	○

CONCEPTION Study - Postpartum Questionnaire 18 months

Overall

Do you think your child hears well?

- Yes
- No

If no, please explain:

Do you think your child talks like other toddlers his age?

- Yes
- No

If no, please explain:

Can you understand most of what your child says?

- Yes
- No

If no, please explain:

Do you think your child walks, runs, and climbs like other toddlers their age?

- Yes
- No

If no, please explain:

Does either parent have a family history of childhood deafness or hearing impairment?

- Yes
- No

If yes, please explain:

Do you have concerns about your child's vision?

- Yes
- No

If yes, please explain:

Do you have any concerns about your child's behaviour?

- Yes
- No

If yes, please explain:

Does anything about your child worry you?

- Yes
- No

If yes, please explain:

Has your child had any medical problems in the last several months?

- Yes
- No

If yes, please explain:

CONCEPTION Study - Postpartum Questionnaire 18 months

Your child's sleeping habits

In this section, we will ask you a few questions about your child's sleeping habits and patterns.

At night, how many consecutive hours does your child sleep without waking up?

How often does your baby usually wake up each night?

- Almost never
- Normally once during the night
- 2 times during the night
- 3 times during the night
- More than 3 times
- Don't know

Usually, how many naps does your baby take during the day?

- Less than 2 naps during the day
- 2 naps during the day
- 3 naps during the day
- More than 3 naps during the day
- Don't know

Depending on their values and preferences, families choose different types of arrangements for their baby's sleep. Generally speaking, where does your baby sleep at night?

- ◯ My child sleeps alone in a room
- ◯ My child sleeps in my room, but in his own bed
- ◯ My child sleeps in my room and shares my bed
- ◯ My child sleeps in his bed part of the night and in my bed another part of the night
- ◯ Don't know
- ◯ Other (please specify)

How satisfied are you with where your baby is currently sleeping?

- ◯ Not at all satisfied
- ◯ Quite satisfied
- ◯ Very satisfied
- ◯ Don't know

Does your child exhibit these behaviors while sleeping:

	Never	Sometimes	Often	Always	N/A
Noisy breathing	○	○	○	○	○
Breathing pauses (apnea)	○	○	○	○	○
Night terrors (i.e. waking up suddenly with intense screams and crying, sometimes sweaty and appears inconsolable for several minutes, even when taken)	○	○	○	○	○
Teeth grinding (bruxism)	○	○	○	○	○
Sleepwalking (i.e walking while sleeping)	○	○	○	○	○
Nocturnal rhythms (do they rock themselves or repeatedly hit their head against his bed or pillow before falling asleep or while sleeping?)	○	○	○	○	○

CONCEPTION Study - Postpartum Questionnaire 18 months

Infant/toddler sensory profile

In this final section, we will be assessing the sensory profile of your child.

Please answer the following questions based on what describes your child best using the following scale :
Almost always: if subjected to this situation, your child almost always shows this behaviour **(90% of the time)**
Frequently: if subjected to this situation, your child frequently shows this behaviour **(75% of the time)**
Occasionally: if subjected to this situation, your child occasionally shows this behaviour **(50% of the time)**
Rarely: if subjected to this situation, your child rarely shows this behaviour **(25% of the time)**
Almost never : if subjected to this situation, your child rarely shows this behaviour **(10% of the time)**

Auditory Processing

	Almost always	Frequently	Occasionally	Rarely	Almost never
I have to speak loudly to get my child's attention	◯	◯	◯	◯	◯
I have to touch my child to gain attention	◯	◯	◯	◯	◯
My child enjoys making sounds with his/her mouth	◯	◯	◯	◯	◯
My child takes a long time to respond, even to familiar voices	◯	◯	◯	◯	◯
My child startles easily at sound, compared to other children the same age	◯	◯	◯	◯	◯
My child is distracted and/or has difficulty eating in noisy environments	◯	◯	◯	◯	◯
My child ignores me when I am talking	◯	◯	◯	◯	◯
My child tries to escape from noisy environments	◯	◯	◯	◯	◯
My child finds ways to make noise with toys	◯	◯	◯	◯	◯
It takes a long time for my child to respond to his/her name when it is called	◯	◯	◯	◯	◯

Visual processing

	Almost always	Frequently	Occasionally	Rarely	Almost never
My child enjoys looking at moving or spinning objects (for example, ceiling fans, toys with wheels, floor fans)	◯	◯	◯	◯	◯
My child enjoys looking at shiny objects	◯	◯	◯	◯	◯
My child avoids eye contact with me	◯	◯	◯	◯	◯
My child refuses to look at books with me	◯	◯	◯	◯	◯
My child does not recognize self in the mirror	◯	◯	◯	◯	◯
My child enjoys looking at own reflection in the mirror	◯	◯	◯	◯	◯
My child prefers fast-paced, brightly colored TV shows	◯	◯	◯	◯	◯

Avoidance

	Almost always	Frequently	Occasionally	Rarely	Almost never
My child resists playing among other children	◯	◯	◯	◯	◯
My child gets anxious in new situations	◯	◯	◯	◯	◯
My child resists cuddled	◯	◯	◯	◯	◯
My child is upset when moving among spaces	◯	◯	◯	◯	◯

with very different temperatures (for example, colder, warmer)	◯	◯	◯	◯	◯
My child withdraws from contact with rough, cold, or sticky surfaces (for example, carpet, countertops)	◯	◯	◯	◯	◯
My child becomes upset if own clothing, hands, or face are messy	◯	◯	◯	◯	◯
My child withdraws from unexpected touch	◯	◯	◯	◯	◯
My child shows a clear dislike for all but a few food choices	◯	◯	◯	◯	◯
My child has temper tantrums	◯	◯	◯	◯	◯
My child is bothered by new settings	◯	◯	◯	◯	◯
My child becomes so upset in new settings that it's hard to calm down	◯	◯	◯	◯	◯

CONCEPTION Study - Postpartum Questionnaire 18 months

Thank you so much for your time!

You have now finished. Thank you for answering this questionnaire. We assure you that this data will remain confidential. If you have questions, do not hesitate to ask the study coordinator for help.

Jessica Gorgui, M.Sc.
Study coordinator
jessica.gorgui.hsj@ssss.gouv.qc.ca
514-345-4931 ext. 7478
1-866-220-2654 (toll-free)
