# Supplementary material for: Maternal Mental Health in Pregnancy and Its Impact on Children’s Cognitive Development at 18 Months, during the COVID-19 Pandemic (CONCEPTION Study)
Source: J Clin Med. 2024 Feb 13;13(4):1055. doi: 10.3390/jcm13041055 (PMC10889100; doi:10.3390/jcm13041055)
Supplement: Supplementary file 1 [file jcm-13-01055-s001.zip › Sup_Files_CONCEPTION_18_MO.pdf]

Table 1A Association between *in utero* exposure to depression, anxiety, stress, antidepressants, and Ages and stages questionnaire – third edition (ASQ-3) - cohort restricted to children aged 17 to 19 months

	DS	RLAM	Crude OR (95%CI)	Adjusted OR* (95%CI)	FRAP	Crude OR (95%CI)	Adjusted OR* (95% CI)
				**Communication**			
***In utero* exposure to maternal depression**							
None to mild (EPDS<9)	173 (60.5)	33 (57.9)	Reference	Reference	3 (50.0)	Reference	Reference
Moderate to severe (EPDS≥ 9)	113 (39.5)	24 (42.1)	1.11 (0.63;1.98)	0.98 (0.48;2.02)	3 (50.0)	1.53 (0.30;7.72)	1.60 (0.16;16.0)
***In utero* exposure to maternal anxiety**							
None to mild (GAD-7 ≤ 9)	263 (92.0)	50 (87.7)	Reference	Reference	4.0 (66.7)	Reference	Reference
Moderate to severe (GAD-7>9)	23 (8.0)	7.0 (12.3)	1.60 (0.65;3.93)	1.70 (0.56;5.13)	2 (33.3)	5.72 (0.99;32.9)	7.59 (0.41;140)
***In utero* exposure to stress related to the COVID-19 pandemic;mean (SD)**	4.4 (2.1)	4.6 (2.0)	1.03 (0.90;1.18)	1.03 (0.87;1.23)	4.3 (2.4)	0.97 (0.66;1.44)	0.93 (0.53;1.62)
***In utero* exposure to maternal antidepressants**							
No	229 (80.1)	41 (71.9)			4 (66.7)		
Yes	57 (19.9)	16 (28.1)	1.57 (0.82;2.99)	2.05 (0.66;6.32)	2 (33.3)	2.01 (0.36;11.2)	0.45 (0.01;20.6)
				**Gross motor**			
***In utero* exposure to maternal depression**							
None to mild (EPDS<9)	175 (59.7)	20 (60.6)	Reference	Reference	12 (54.5)	Reference	Reference
Moderate to severe (EPDS≥ 9)	118 (40.3)	13 (39.4)	0.96 (0.46;2.01)	0.82 (0.29;2.29)	10 (45.5)	1.24 (0.52;2.95)	0.78 (0.26;2.34)
***In utero* exposure to maternal anxiety**							
None to mild (GAD-7 ≤ 9)	269 (91.8)	26 (78.8)	Reference	Reference	20 (90.9)	Reference	Reference
Moderate to severe (GAD-7>9)	24 (8.2)	7 (21.2)	3.02 (1.19;7.67)	**5.53 (1.52;20.1)**	2 (9.1)	1.12 (0.25;5.09)	1.00 (0.15;6.62)
***In utero* exposure to stress related to the COVID-19 pandemic;mean (SD)**	4.5 (2.1)	4.1 (1.7)	0.91 (0.76;1.08)	0.84 (0.66;1.06)	5.2 (1.7)	1.18 (0.96;1.47)	1.31 (0.99;1.73)
***In utero* exposure to maternal antidepressants**							
No	232 (79.2)	23 (69.7)	Reference	Reference	19 (86.4)	Reference	Reference
Yes	61 (20.8)	10 (30.3)	1.65 (0.75;3.66)	3.09 (0.67;14.2)	3 (13.6)	0.60 (0.17;2.10)	0.79 (0.10;6.30)

Table 1A (Continued)

	DS	RLAM	Crude OR (95%CI)	Adjusted OR* (95%CI)	FRAP	Crude OR (95%CI)	Adjusted OR* (95% CI)
**Fine motor**							
***In utero* exposure to maternal depression**							
None to mild (EPDS<9)	189 (59.2)	12 (54.5)	Reference	Reference	6 (85.7)	Reference	Reference
Moderate to severe (EPDS≥ 9)	130 (40.8)	10 (45.5)	1.21 (0.51;2.89)	2.21 (0.70;6.97)	1 (14.3)	0.24 (0.03;2.04)	0.10 (0.00;2.56)
***In utero* exposure to maternal anxiety**							
None to mild (GAD-7 ≤ 9)	289 (90.6)	20 (90.9)	Reference	Reference	6 (85.7)	Reference	Reference
Moderate to severe (GAD-7>9)	30 (9.4)	2 (9.1)	0.96 (0.21;4.32)	0.88 (0.15;5.19)	1 (14.3)	1.61 (0.19;13.8)	8.52 (0.30;243)
***In utero* exposure to stress related to the COVID-19 pandemic;mean (SD)**	4.5 (2.1)	4 (1.6)	0.85 (0.69;1.06)	0.78 (0.58;1.05)	3.9 (2.0)	0.88 (0.61;1.28)	0.94 (0.59;1.50)
***In utero* exposure to maternal antidepressants**							
No	253 (79.3)	15 (68.2)	Reference	Reference	6 (85.7)	Reference	Reference
Yes	66 (20.7)	7 (31.8)	1.79 (0.70;4.57)	**4.70 (1.15;19.1)**	1 (14.3)	0.64 (0.08;5.40)	0.39 (0.01;11.8)
**Problem solving**							
***In utero* exposure to maternal depression**							
None to mild (EPDS<9)	160 (57.3)	28 (71.8)	Reference	Reference	16 (72.7)	Reference	Reference
Moderate to severe (EPDS≥ 9)	119 (42.7)	11 (28.2)	0.53 (0.25;1.10)	0.45 (0.18;1.12)	6 (27.3)	0.50 (0.19;1.33)	0.44 (0.13;1.50)
***In utero* exposure to maternal anxiety**							
None to mild (GAD-7 ≤ 9)	251 (90.0)	36 (92.3)	Reference	Reference	21 (95.5)	Reference	Reference
Moderate to severe (GAD-7>9)	28 (10.0)	3 (7.7)	0.75 (0.22;2.58)	1.01 (0.23;4.51)	1 (4.5)	0.43 (0.06;3.30)	0.26 (0.02;3.31)
***In utero* exposure to stress related to the COVID-19 pandemic;mean (SD)**	4.5 (2.1)	4.4 (1.9)	0.98 (0.84;1.16)	1.12 (0.91;1.38)	4.1 (1.8)	0.92 (0.74;1.13)	0.95 (0.73;1.25)
***In utero* exposure to maternal antidepressants**							
No	216 (77.4)	34 (87.2)	Reference	Reference	18 (81.8)	Reference	Reference
Yes	63 (22.6)	5 (12.8)	0.50 (0.19;1.34)	0.65 (0.12;3.40)	4 (18.2)	0.76 (0.25;2.33)	1.04 (0.13;7.98)

Table 1A (Continued)

	DS	RLAM	Crude OR (95%CI)	Adjusted OR* (95%CI)	FRAP	Crude OR (95%CI)	Adjusted OR* (95% CI)
			**Personal and social**				
***In utero* exposure to maternal depression**							
None to mild (EPDS<9)	181 (59.5)	17 (56.7)	Reference	Reference	7 (77.8)	Reference	Reference
Moderate to severe (EPDS≥ 9)	123 (40.5)	13 (43.3)	0.42 (0.09;2.06)	0.34 (0.05;2.26)	2 (22.2)	1.13 (0.53;2.40)	1.29 (0.51;3.24)
***In utero* exposure to maternal anxiety**							
None to mild (GAD-7 ≤ 9)	276 (90.8)	26 (86.7)	Reference	Reference	8 (88.9)	Reference	Reference
Moderate to severe (GAD-7>9)	28 (9.2)	4 (13.3)	1.23 (0.15;10.2)	1.44 (0.11;18.8)	1 (11.1)	1.52 (0.49;4.66)	1.95 (0.53;7.10)
***In utero* exposure to stress related to the COVID-19 pandemic;mean (SD)**	4.5 (2.1)	4.1 (1.7)	1.01 (0.73;1.39)	1.12 (0.73;1.72)	4.6 (1.8)	0.91 (0.76;1.10)	0.87 (0.69;1.09)

SD;standard deviation; DS;Development on schedule; RLAM;Required learning activities and monitoring; FAR;further assessment required by professional

Communication scores categorized: DS above the cut-off;the child appears to be on schedule (30-60); RLAM close to the cut-off;the child needs to be provided with learning activities (15-29); FARP below the cut-off;the child may be needs further assessment by a professional (0 – 14)

Gross motor scores categorized: DS above the cut-off;the child appears to be on schedule (46-60); RLAM close to the cut-off;the child needs to be provided with learning activities (36-45); FARP below the cut-off;the child may be needs further assessment by a professional (0-35)

Fine motor scores categorized: DS above the cut-off;the child appears to be on schedule (45-60); RLAM close to the cut-off;the child needs to be provided with learning activities (35-44); FARP below the cut-off;the child may be needs further assessment by a professional (0-34)

Problem solving scores categorized: DS above the cut-off;the child appears to be on schedule (36-60); RLAM close to the cut-off;the child needs to be provided with learning activities (26-35); FARP below the cut-off;the child may be needs further assessment by a professional (0-25)

Personal and social scores categorized: DS above the cut-off;the child appears to be on schedule (38-60); RLAM close to the cut-off;the child needs to be provided with learning activities (27-37); FARP below the cut-off;the child may be needs further assessment by a professional (0-26)

Using the Edinburgh postnatal depression scale cut-off as follows: scale 0 to 8 means no in utero exposure to maternal depression;scale 9 and more means in utero exposure to moderate to severe maternal depression

Using the generalized anxiety disorder 7-items scale cut-off as follows: scale 0 to 9 means no maternal anxiety;scale 10 and more means in utero exposure to moderate to severe maternal anxiety

Using the overall maternal stress related to COVID-19 measured on a scale from 0 (no stress) to 10 (extreme stress)

Antidepressants use defined by diagnosis of depression or anxiety combined to the use of antidepressants before delivery

*Adjusted models for potential condounders

Communication : maternal depression;maternal age at recruitment;pre-pregnancy body mass index;diabetes (including gestational diabetes);education years;maternal anxiety;maternal stress related to COVID-19 pandemic;period of recruitment;gestational age at delivery;CASC-150 score

Gross motor : maternal depression;maternal age at recruitment;annual household income;pre-pregnancy body mass index;diabetes (including gestational diabetes);hypertension (including preeclampsia);education years;maternal anxiety;maternal stress related to COVID-19 pandemic;period of recruitment;gestational age at delivery;CASC-150 score;medication use;area of residence;maternal asthma;historic of depression;ethnicity;historic of anxiety

Fine motor : maternal depression;maternal age at recruitment;annual household income;pre-pregnancy body mass index;diabetes (including gestational diabetes);education years;maternal anxiety;maternal stress related to COVID-19 pandemic;period of recruitment;gestational age at delivery;CASC-150 score;medication use;area of residence;marital status;historic of anxiety;ethnicity

Problem solving : maternal depression;maternal age at recruitment;annual household income;pre-pregnancy body mass index;diabetes (including gestational diabetes);hypertension (including preeclampsia);education years;maternal anxiety;maternal stress related to COVID-19 pandemic;period of recruitment;gestational age at delivery;CASC-150 score;medication use;maternal asthma;historic of depression;historic of anxiety;ethnicity

Personal and social : maternal depression;maternal age at recruitment;annual household income;pre-pregnancy body mass index;diabetes (including gestational diabetes);hypertension (including preeclampsia);education years;maternal anxiety;maternal stress related to COVID-19 pandemic;period of recruitment;gestational age at delivery;CASC-150 score;medication use;area of residence;marital status;maternal asthma;ethnicity;historic of depression;historic of anxiety

Bold numbers indicate significant association ($p < 0.05$)

Table 2A Association between in utero exposure to depression, anxiety, stress, antidepressants, and Ages and stages questionnaire – third edition (ASQ-3) – stratified on child sex

	Boys				Girls			
	**DS / RLAM**	**FRAP**	**Crude OR (95%CI)**	**Adjusted OR (95%CI)**	**DS / RLAM**	**FRAP**	**Crude OR (95%CI)**	**Adjusted OR (95%CI)**
**Communication[a]**								
***In utero* exposure to maternal depression**								
None to mild (EPDS<9)	131 (63.0)	4 (50.0)	Reference	Reference	106 (53.3)	1 (50.0)	Reference	Reference
Moderate to severe (EPDS≥ 9)	77 (37.0)	4 (50.0)	1.70 (0.39;7.38)	1.63 (0.19;14.1)	93 (46.7)	1 (50.0)	1.14 (0.04;29.1)	0.01 (0.00;4.08)
***In utero* exposure to maternal anxiety**								
None to mild (GAD-7 ≤ 9)	188 (90.4)	6 (75.0)	Reference	Reference	182 (91.5%)	1 (50.0%)	Reference	Reference
Moderate to severe (GAD-7>9)	20 (9.6)	2 (25.0)	3.13 (0.44;14.7)	4.71 (0.20;121)	17 (8.5%)	1 (50.0%)	10.7 (0.41;279)	NA
***In utero* exposure to stress related to the COVID-19 pandemic;mean (SD)**	4.3 (2.2)	4.9 (2.4)	1.12 (0.81;1.57)	1.23 (0.72;2.15)	4.7 (2.0)	7 (2.8)	2.31 (0.95;8.53)	2.51 (0.64;31.7)
***In utero* exposure to maternal antidepressants**								
No	173 (83.2)	6 (75.0)	Reference	Reference	151 (75.9%)	2 (100.0%)	NA	NA
Yes	35 (16.8)	2 (25.0)	1.65 (0.23;7.50)	0.31 (0.01;5.67)	48 (24.1%)	0 (0.0%)	NA	NA
**Gross motor[b]**								
***In utero* exposure to maternal depression**								
None to mild (EPDS<9)	128 (64.7)	7 (38.9)	Reference	Reference	93 (51.1)	12 (66.7)	Reference	Reference
Moderate to severe (EPDS≥ 9)	70 (35.4)	11 (61.1)	**2.87 (1.08;8.12)**	1.01 (0.87;1.18)	89 (48.9)	6 (33.3)	0.52 (0.18;1.41)	**0.18 (0.03;0.73)**

Table 2A (Continued)

	Boys				Girls			
	**DS / RLAM**	**FRAP**	**Crude OR (95%CI)**	**Adjusted OR (95%CI)**	**DS / RLAM**	**FRAP**	**Crude OR (95%CI)**	**Adjusted OR (95%CI)**
***In utero* exposure to maternal anxiety**								
None to mild (GAD-7 ≤ 9)	178 (89.9)	16 (88.9)	Reference	Reference	166 (91.2)	15 (83.3)	Reference	Reference
Moderate to severe (GAD-7>9)	20 (10.1)	2 (11.1)	1.11 (0.17;4.31)	1.24 (0.12;9.87)	16 (8.8)	3 (16.7)	2.08 (0.45;7.16)	2.68 (0.37;19.5)
***In utero* exposure to stress related to the COVID-19 pandemic, mean (SD)**	4.3 (2.2)	4.7 (2.2)	1.45 (0.98;2.31)	1.53 (0.86;3.18)	4.7 (2.0)	4.6 (2.0)	1.18 (0.92;1.55)	**1.44 (1.04;2.06)**
***In utero* exposure to maternal antidepressants**			1.08 (0.86;1.35)	1.18 (0.85;1.68)				
No	165 (83.3)	14 (77.8)	Reference	Reference	139 (76.4)	14 (77.8)	Reference	Reference
Yes	33 (16.7)	4 (22.2)	1.43 (0.39;4.28)	0.53 (0.03;5.93)	43 (23.6)	4 (22.2)	0.92 (0.25;2.73)	4.02 (0.46;37.1)
**Fine motor[c]**								
***In utero* exposure to maternal depression**								
None to mild (EPDS<9)	130 (62.2)	5 (71.4)	Reference	Reference	103 (52.6)	2 (50.0)	Reference	Reference
Moderate to severe (EPDS≥ 9)	79 (37.8)	2 (28.6)	0.66 (0.09;3.13)	0.63 (0.05;5.13)	93 (47.4)	2 (50.0)	1.11 (0.13;9.38)	0.31 (0.01;7.86)
***In utero* exposure to maternal anxiety**								
None to mild (GAD-7 ≤ 9)	188 (90)	6 (85.7)	Reference	Reference	179 (91.3)	2 (50.0)	Reference	Reference
Moderate to severe (GAD-7>9)	21 (10)	1 (14.3)	1.49 (0.08;9.33)	3.02 (0.05;98.2)	17 (8.7)	2 (50.0)	10.5 (1.20;92.4)	NA

Table 2A (Continued)

	Boys				Girls			
	**DS / RLAM**	**FRAP**	**Crude OR (95%CI)**	**Adjusted OR (95%CI)**	**DS / RLAM**	**FRAP**	**Crude OR (95%CI)**	**Adjusted OR (95%CI)**
***In utero* exposure to stress related to the COVID-19 pandemic, mean (SD)**	4.3 (2.2)	4.7 (2)	1.08 (0.76;1.54)	1.25 (0.77;2.14)	4.7 (2.0)	5.0 (3.4)	1.08 (0.66;1.88)	0.85 (0.39;1.73)
***In utero* exposure to maternal antidepressants**								
No	173 (82.8)	6 (85.7)	Reference	Reference	150 (76.5)	3 (75.0)	Reference	Reference
Yes	36 (17.2)	1 (14.3)	0.80 (0.04;4.89)	0.37 (0.01;6.06)	46 (23.5)	1 (25.0)	1.09 (0.05;8.72)	0.45 (0.00;36.3)
**Problem solving[d]**								
***In utero* exposure to maternal depression**								
None to mild (EPDS<9)	118 (62.1)	16 (72.7)	Reference	Reference	99 (53.2)	4 (50.0)	Reference	Reference
Moderate to severe (EPDS≥ 9)	72 (37.9)	6 (27.3)	0.61 (0.21;1.57)	0.70 (0.19;2.32)	87 (46.8)	4 (50.0)	1.14 (0.26;4.94)	0.26 (0.02;2.16)
***In utero* exposure to maternal anxiety**								
None to mild (GAD-7 ≤ 9)	169 (88.9)	21 (95.5)	Reference	Reference	171 (91.9)	6 (75.0)	Reference	Reference
Moderate to severe (GAD-7>9)	21 (11.1)	1 (4.6)	0.38 (0.02;1.99)	0.44 (0.02;3.32)	15 (8.1)	2 (25.0)	3.80 (0.53;18.2)	**21.8 (1.05;846)**
***In utero* exposure to stress related to the COVID-19 pandemic, mean (SD)**	4.4 (2.2)	4 (2.2)	0.91 (0.74;1.12)	0.91 (0.69;1.19)	4.6 (1.9)	5.9 (2.1)	1.45 (0.98;2.31)	1.53 (0.86;3.18)
***In utero* exposure to maternal antidepressants**								
No	157 (82.6)	18 (81.8)	Reference	Reference	142 (76.3)	7 (87.5)	Reference	Reference
Yes	33 (17.4)	4 (18.2)	1.06 (0.29;3.06)	2.03 (0.37;9.16)	44 (23.7)	1 (12.5)	0.46 (0.02;2.69)	0.09 (0.00;5.39)

Table 2A (Continued)

	Boys				Girls			
	**DS / RLAM**	**FRAP**	**Crude OR (95%CI)**	**Adjusted OR (95%CI)**	**DS / RLAM**	**FRAP**	**Crude OR (95%CI)**	**Adjusted OR (95%CI)**
**Personal and social[e]**								
***In utero* exposure to maternal depression**								
None to mild (EPDS<9)	128 (61.54)	7 (100.00)	NA	NA	100 (52.9)	3 (50.0)	Reference	Reference
Moderate to severe (EPDS≥ 9)	80 (38.46)	0 (0.00)	NA	NA	89 (47.1)	3 (50.0)	1.12 (0.20;6.20)	0.57 (0.05;5.03)
***In utero* exposure to maternal anxiety**								
None to mild (GAD-7 ≤ 9)	187 (89.9)	6 (85.7)	Reference	Reference	172 (91.0)	5 (83.3)	Reference	Reference
Moderate to severe (GAD-7>9)	21 (10.1)	1 (14.3)	1.48 (0.08;9.28)	2.40 (0.08;34.4)	17 (9.0)	1 (16.7)	2.02 (0.10;13.5)	2.39 (0.09;36.7)
***In utero* exposure to stress related to the COVID-19 pandemic, mean (SD)**	4.4 (2.2)	4.1 (2.3)	0.96 (0.67;1.35)	1.15 (0.67;2.04)	4.7 (2.0)	5.3 (1.0)	1.20 (0.79;1.95)	1.32 (0.73;2.62)

Na;none applicable

SD;standard deviation; DS;Development on schedule; RLAM;Required learning activities and monitoring; FARP;further assessment required by professional

Communication scores categorized: DS/ RLAM;the child appears to be on schedule (14-60); FARP (0 – 14); Gross motor scores categorized: DS/ RLAM;the child appears to be on schedule (36 60); FARP;the child may be needs further assessment by a professional (0-35); Fine motor scores categorized: DS/ RLAM;the child appears to be on schedule (35-60); FARP;the child may be needs further assessment by a professional (0-34); Problem solving scores categorized: DS/ RLAM;the child appears to be on schedule (26-60); FARP;the child may be needs further assessment by a professional (0-25); Personal and social scores categorized: DS/ RLAM;the child appears to be on schedule (27-60); FARP;;the child may be needs further assessment by a professional (0-26)

Using the Edinburgh postnatal depression scale cut-off as follows: scale 0 to 8 means no in utero exposure to maternal depression;scale 9 and more means in utero exposure to moderate to severe maternal depression

Using the generalized anxiety disorder 7-items scale cut-off as follows: scale 0 to 9 means no maternal anxiety;scale 10 and more means in utero exposure to moderate to severe maternal anxiety

Using the overall maternal stress related to COVID-19 measured on a scale from 0 (no stress) to 10 (extreme stress)

Antidepressants use defined by diagnosis of depression or anxiety combined to the use of antidepressants before delivery

[*]Adjusted models

For boys:

Communication: maternal depression;maternal age;area of residence;pre-pregnancy body mass index;maternal asthma;maternal diabetes (including gestational diabetes);maternal hypertension (including preeclampsia);education years;maternal anxiety;maternal stress related to COVID,19 pandemic;antidepressants use;historic of depression;medication use;ethnicity;period of recruitment;gestational age at recruitment;CASC150 score

Gross motor : maternal depression;maternal age;area of residence;annual household income;marital status;pre-pregnancy body mass index;maternal diabetes (including gestational diabetes);education years;maternal anxiety;maternal stress related to COVID,19 pandemic;antidepressants use;historic of depression;historic of anxiety;period of recruitment;gestational age at recruitment;CASC150 scores;medication use;ethnicity
Fine motor : maternal depression;maternal age;area of residence;annual household income;pre-pregnancy body mass index;maternal asthma;maternal diabetes (including gestational diabetes);education years;maternal anxiety;maternal stress related to COVID,19 pandemic;antidepressants use;ethnicity;gestational age at recruitment;CASC150 scores;medication use
Problem solving : maternal depression;maternal age;area of residence;annual household income;pre-pregnancy body mass index;maternal asthma;maternal diabetes (including gestational diabetes);maternal hypertension (including preeclampsia);education years;maternal anxiety;ethnicity;medication use;maternal stress related to COVID,19 pandemic;antidepressants use;historic of depression;period of recruitment;gestational age at recruitment;CASC150 scores
Personal and social : maternal age;pre-pregnancy body mass index;maternal diabetes (including gestational diabetes);education years;maternal anxiety;maternal stress related to COVID,19 pandemic;period of recruitment;gestational age at recruitment;CASC150 scores;medication use
For girls:
Communication : maternal depression;maternal age;pre-pregnancy body mass index;maternal hypertension (including preeclampsia);education years;maternal stress related to COVID,19 pandemic;gestational age at recruitment;CASC150 score;the generalized anxiety disorder 7-items scale continuous score
Gross motor : maternal depression;maternal age;area of residence;pre-pregnancy body mass index;maternal diabetes (including gestational diabetes);maternal hypertension (including preeclampsia);education years;maternal anxiety;maternal stress related to COVID,19 pandemic;antidepressants use;historic of depression;historic of anxiety;period of recruitment;gestational age at recruitment ;CASC150 score;medication use;ethnicity
Fine motor : maternal depression;maternal age;annual household income;pre-pregnancy body mass index;maternal diabetes (including gestational diabetes);education years;maternal stress related to COVID,19 pandemic;antidepressants use;historic of depression;historic of anxiety;gestational age at recruitment;CASC150 score;medication use;the generalized anxiety disorder 7-items scale continuous score
Problem solving : for maternal depression;maternal age;area of residence;marital status;pre-pregnancy body mass index;maternal asthma;maternal diabetes (including gestational diabetes);education years;maternal anxiety;ethnicity;medication use;maternal stress related to COVID,19 pandemic;antidepressants use;historic of depression;historic of anxiety;gestational age at recruitment;CASC150 score
Personal and social : maternal depression;maternal age;annual household income;pre-pregnancy body mass index;maternal diabetes (including gestational diabetes);maternal hypertension (including preeclampsia);education years;maternal anxiety;maternal stress related to COVID,19 pandemic;gestational age at recruitment;CASC150 score;medication use
Bold numbers indicate significant association ($p < 0.05$)

Table 3A Association between in utero exposure to depression, anxiety, stress, antidepressants, and Ages and stages questionnaire – third edition (ASQ-3) – stratified for child status infection by COVID-19

	Infected by COVID-19				Not infected by COVID-19			
	**DS / RLAM**	**FRAP**	**Crude OR (95%CI)**	**Adjusted OR (95%CI)**	**DS / RLAM**	**FRAP**	**Crude OR (95%CI)**	**Adjusted OR (95%CI)**
				**Communication[a]**				
***In utero* exposure to maternal depression**								
None to mild (EPDS<9)	157 (60.4)	1 (25.0)	Reference	Reference	111 (55.8)	4 (66.7)	Reference	Reference
Moderate to severe (EPDS≥ 9)	103 (39.6)	3 (75.0)	4.57 (0.58;93.1)	12.7 (0.59;681)	88 (44.2)	2 (33.3)	0.63 (0.09;3.31)	**0.05 (0.00;0.85)**
***In utero* exposure to maternal anxiety**								
None to mild (GAD-7 ≤ 9)	240 (92.3)	3 (75.0)	Reference	Reference	177 (88.9)	3 (50)	Reference	Reference
Moderate to severe (GAD-7>9)	20 (7.7)	1 (25.0)	4.00 (0.19;32.9)	2.64 (0.06;82.1)	22 (11.1)	3 (50)	**8.05 (1.42;45.9)**	**86.0 (3.25;11,212)**
***In utero* exposure to stress related to the COVID-19 pandemic, mean (SD)**	4.5 (2.0)	4.8 (2.8)	1.06 (0.65;1.79)	0.69 (0.28;1.46)	4.4 (2.2)	4.8 (2.4)	1.09 (0.75;1.60)	1.42 (0.77;2.91)
***In utero* exposure to maternal antidepressants**								
No	205 (78.8)	3 (75.0)	Reference	Reference	159 (79.9)	5 (83.3)	Reference	Reference
Yes	55 (21.2)	1 (25.0)	1.24 (0.06;9.92)	0.14 (0.00;72.0)	40 (20.1)	1 (16.7)	0.80 (0.04;5.11)	0.52 (0.00;48.5)
				**Gross motor[b]**				
***In utero* exposure to maternal depression**								
None to mild (EPDS<9)	148 (60.7)	8 (44.4)	Reference	Reference	101 (55.2)	14 (60.9)	Reference	Reference
Moderate to severe (EPDS≥ 9)	96 (39.3)	10 (55.6)	1.93 (0.73;5.21)	1.57 (0.45;5.51)	82 (44.8)	9 (39.1)	0.79 (0.32;1.90)	0.78 (0.21;2.73)
***In utero* exposure to maternal anxiety**								
None to mild (GAD-7 ≤ 9)	225 (92.2)	16 (88.9)	Reference	Reference	160 (87.4)	20 (87.0)	Reference	Reference
Moderate to severe (GAD-7>9)	19 (7.8)	2 (11.1)	1.48 (0.22;5.75)	1.27 (0.15;7.39)	23 (12.6)	3 (13.0)	1.04 (0.23;3.36)	0.78 (0.12;4.23)

Table 3A (Continued)

	Infected by COVID-19				Not infected by COVID-19			
	**DS / RLAM**	**FRAP**	**Crude OR (95%CI)**	**Adjusted OR (95%CI)**	**DS / RLAM**	**FRAP**	**Crude OR (95%CI)**	**Adjusted OR (95%CI)**
**In utero exposure to stress related to the COVID-19 pandemic, mean (SD)**	4.5 (2.0)	5.2 (1.9)	1.19 (0.94;1.54)	1.25 (0.91;1.74)	4.4 (2.2)	4.6 (2.2)	1.04 (0.85;1.27)	1.15 (0.87;1.53)
***In utero* exposure to maternal antidepressants**								
No	194 (79.5)	14 (77.8)	Reference	Reference	146 (79.8)	18 (78.3)	Reference	Reference
Yes	50 (20.5)	4 (22.2)	1.11 (0.30;3.25)	0.32 (0.02;3.84)	37 (20.2)	5 (21.7)	1.10 (0.34;2.96)	1.90 (0.33;10.9)
				**Fine motor[c]**				
***In utero* exposure to maternal depression**								
None to mild (EPDS<9)	154 (59.5)	2 (66.7)	Reference	Reference	109 (55.1)	6 (75.0)	Reference	Reference
Moderate to severe (EPDS≥ 9)	105 (40.5)	1 (33.3)	0.73 (0.03;7.75)	7.80 (0.20;443)	89 (44.9)	2 (25.0)	0.41 (0.06;1.82)	**0.02 (0.00;0.38)**
**Stress in utero related to COVID-19 pandemic, mean (SD)**	4.6 (2.0)	2 (1.0)	**0.47 (0.19;0.91)**	**0.38 (0.10;0.92)**	4.4 (2.2)	5.4 (1.7)	1.23 (0.89;1.74)	**1.73 (1.01;3.35)**
**Maternal antidepressants use**								
No	205 (79.2)	3 (100.0)	NA	NA	158 (79.8)	6 (75.0)	Reference	Reference
Yes	54 (20.8)	0 (0.0)	NA	NA	40 (20.2)	2 (25.0)	1.32 (0.19;5.96)	0.84 (0.02;19.8)
				**Problem solving**				
***In utero* exposure to maternal depression**								
None to mild (EPDS<9)	118 (62.1)	16 (72.7)	Reference	Reference	105 (56.5)	8 (61.5)	Reference	Reference
Moderate to severe (EPDS≥ 9)	72 (37.9)	6 (27.3)	0.61 (0.21;1.57)	0.70 (0.19;2.32)	81 (43.5)	5 (38.5)	0.81 (0.24;2.52)	0.36 (0.05;1.91)
***In utero* exposure to maternal anxiety**								
None to mild (GAD-7 ≤ 9)	169 (88.9)	21 (95.5)	Reference	Reference	165 (88.7)	10 (76.9)	Reference	Reference
Moderate to severe (GAD-7>9)	21 (11.1)	1 (4.6)	0.38 (0.02;1.99)	0.44 (0.02;3.32)	21 (11.3)	3 (23.1)	2.36 (0.50;8.45)	6.17 (0.65;66.9)
***In utero* exposure to stress related to the COVID-19 pandemic, mean (SD)**	4.4 (2.2)	4 (2.2)			4.4 (2.2)	4.6 (2.4)	1.05 (0.81;1.36)	1.02 (0.69;1.51)

Table 3A (Continued)

	Boys				Girls			
	**DS / RLAM**	**FRAP**	**Crude OR (95%CI)**	**Adjusted OR (95%CI)**	**DS / RLAM**	**FRAP**	**Crude OR (95%CI)**	**Adjusted OR (95%CI)**
No	157 (82.6)	18 (81.8)	Reference	Reference	148 (79.6)	10 (76.9)	Reference	Reference
Yes	33 (17.4)	(18.2)	1.06 (0.29;3.06)	2.03 (0.37;9.16)	38 (20.4)	3 (23.1)	1.17 (0.25;4.04)	0.43 (0.03;3.94)
**Personal and social[e]**								
***In utero* exposure to maternal depression**								
None to mild (EPDS<9)	148 (59.0)	6 (85.7)	Reference	Reference	110 (56.1)	4 (66.7)	Reference	Reference
Moderate to severe (EPDS≥ 9)	103 (41.0)	1 (14.3)	0.24 (0.01;1.43)	0.08 (0.00;1.16)	86 (43.9)	2 (33.3)	0.64 (0.09;3.36)	0.27 (0.01;2.53)
***In utero* exposure to maternal anxiety**								
None to mild (GAD-7 ≤ 9)	231 (92.0)	6 (85.7)	Reference	Reference	172 (87.8)	5 (83.3)	Reference	Reference
Moderate to severe (GAD-7>9)	20 (8.0)	1 (14.3)	1.92 (0.10;12.1)	1.53 (0.03;87.5)	24 (12.2)	1 (16.7)	1.43 (0.07;9.40)	3.93 (0.11;103)
***In utero* exposure to stress related to the COVID-19 pandemic, mean (SD)**	4.5 (2.0)	4.6 (2.1)	1.01 (0.69;1.49)	1.23 (0.70;2.21)	4.4 (2.2)	4.8 (1.7)	1.09 (0.75;1.61)	1.40 (0.81;2.59)

Na;none applicable

SD;standard deviation; DS;Development on schedule; RLAM;Required learning activities and monitoring; FAR;further assessment required by professional

Communication scores categorized: DS/ RLAM;the child appears to be on schedule (14-60); FARP (0 – 14); Gross motor scores categorized: DS/ RLAM;the child appears to be on schedule (36 60); FARP;the child may be needs further assessment by a professional (0-35); Fine motor scores categorized: DS/ RLAM;the child appears to be on schedule (35-60); FARP;the child may be needs further assessment by a professional (0-34); Problem solving scores categorized: DS/ RLAM;the child appears to be on schedule (26-60); FARP;the child may be needs further assessment by a professional (0-25); Personal and social scores categorized: DS/ RLAM;the child appears to be on schedule (27-60); FARP;;the child may be needs further assessment by a professional (0-26)

Using the Edinburgh postnatal depression scale cut-off as follows: scale 0 to 8 means no in utero exposure to maternal depression;scale 9 and more means in utero exposure to moderate to severe maternal depression

Using the generalized anxiety disorder 7-items scale cut-off as follows: scale 0 to 9 means no maternal anxiety;scale 10 and more means in utero exposure to moderate to severe maternal anxiety

Using the overall maternal stress related to COVID-19 measured on a scale from 0 (no stress) to 10 (extreme stress)

Antidepressants use defined by diagnosis of depression or anxiety combined to the use of antidepressants before delivery

Adjusted models

For children infected by COVID-19

Communication : maternal depression;maternal age;pre-pregnancy body mass index;maternal asthma;education year;maternal anxiety;maternal stress;antidepressants use;historic of depression;ethnicity;historic of anxiety;period of recruitment;gestational age at recruitment;CONCEPTION study Assessment of Stress from COVID-19 – 150 points score (CASC150 score)

Gross motor : maternal depression;maternal age;area of residence;annual household income;marital status;pre-pregnancy body mass index ;education year;maternal anxiety;maternal stress Antidepressants use;historic of depression;historic of anxiety;period of recruitment;gestational age at recruitment;CONCEPTION study Assessment of Stress from COVID-19 – 150 points score;medication use

Fine motor : maternal depression;maternal age;pre-pregnancy body mass index;education year;maternal stress;gestational age at recruitment;CASC150 score;medication use

Problem solving : maternal depression;maternal age;area of residence;annual household income;pre-pregnancy body mass index;maternal asthma;maternal diabetes (including gestational diabetes);education year;maternal anxiety;ethnicity;medication use;maternal stress;antidepressants use;historic of depression;historic of anxiety;period of recruitment;gestational age at recruitment;CASC150 score

Personal and social : maternal depression;maternal age;annual household income;pre-pregnancy body mass index;maternal diabetes (including gestational diabetes);maternal hypertension (including preeclampsia);education year;maternal anxiety;maternal stress;period of recruitment;gestational age at recruitment;CASC150 score;medication use

For children not infected by COVID-19

Communication : maternal anxiety;maternal age;pre-pregnancy body mass index;maternal asthma;maternal diabetes (including gestational diabetes);maternal hypertension (including preeclampsia);education year;the Edinburgh postnatal depression scale continuously;maternal stress;antidepressants use;medication use;ethnicity;historic of anxiety;gestational age at recruitment;CASC150 score

Gros motor : maternal depression;maternal age;area of residence;annual household income;marital status;pre-pregnancy body mass index;maternal diabetes (including gestational diabetes);maternal hypertension (including preeclampsia);education year;maternal anxiety;maternal stress;antidepressants use;historic of depression;historic of anxiety;period of recruitment;gestational age at recruitment;CASC150 score;medication use;ethnicity

Fine motor : maternal anxiety;maternal age;area of residence;annual household income;pre-pregnancy body mass index;maternal asthma;maternal diabetes (including gestational diabetes);education year;EPDStotal1;maternal stress; antidepressants use;historic of depression;historic of anxiety;period of recruitment;ethnicity;gestational age at recruitment;CASC150 score;medication use

Problem solving : maternal depression;maternal age;area of residence;annual household income;marital status;pre-pregnancy body mass index;maternal diabetes (including gestational diabetes);maternal hypertension (including preeclampsia);education year;maternal anxiety;ethnicity;medication use;maternal stress;antidepressants use;historic of depression;historic of anxiety;gestational age at recruitment;CASC150 score

Personal and social : maternal depression;maternal age;annual household income;pre-pregnancy body mass index;maternal diabetes (including gestational diabetes);education year;maternal anxiety;maternal stress;gestational age at recruitment;CASC150 score;medication use
